# Lignocellulosic Materials for the Production of Biofuels, Biochemicals and Biomaterials and Applications of Lignocellulose-Based Polyurethanes: A Review

**DOI:** 10.3390/polym14050881

**Published:** 2022-02-23

**Authors:** Antonio M. Borrero-López, Concepción Valencia, José M. Franco

**Affiliations:** Pro2TecS—Chemical Process and Product Technology Research Center, Departamento de Ingeniería Química, Escuela Técnica Superior de Ingeniería, Campus de “El Carmen”, Universidad de Huelva, 21071 Huelva, Spain; barragan@uhu.es (C.V.); franco@uhu.es (J.M.F.)

**Keywords:** lignocellulose, lignin, cellulose, biofuels, biomaterials, biochemicals, lubricating greases, adhesives, cushioning materials, rheology

## Abstract

The present review is devoted to the description of the state-of-the-art techniques and procedures concerning treatments and modifications of lignocellulosic materials in order to use them as precursors for biomaterials, biochemicals and biofuels, with particular focus on lignin and lignin-based products. Four different main pretreatment types are outlined, i.e., thermal, mechanical, chemical and biological, with special emphasis on the biological action of fungi and bacteria. Therefore, by selecting a determined type of fungi or bacteria, some of the fractions may remain unaltered, while others may be decomposed. In this sense, the possibilities to obtain different final products are massive, depending on the type of microorganism and the biomass selected. Biofuels, biochemicals and biomaterials derived from lignocellulose are extensively described, covering those obtained from the lignocellulose as a whole, but also from the main biopolymers that comprise its structure, i.e., cellulose, hemicellulose and lignin. In addition, special attention has been paid to the formulation of bio-polyurethanes from lignocellulosic materials, focusing more specifically on their applications in the lubricant, adhesive and cushioning material fields. High-performance alternatives to petroleum-derived products have been reported, such as adhesives that substantially exceed the adhesion performance of those commercially available in different surfaces, lubricating greases with tribological behaviour superior to those in lithium and calcium soap and elastomers with excellent static and dynamic performance.

## 1. Lignocellulose as Renewable Raw Material: Sources and General Chemical Description

It is well known that with the increasing issues of climate change, waste management and unstoppable resource exhaustion, politics and research efforts need to be combined in the search for new materials and sources that can replace fossil fuels and non-renewable resources currently in use, which besides generally include hazardous/toxic manufacture protocols and problematic end-of-life. The anthropogenic imprint on global temperature has already been reported as an increase of 0.87 °C, and it is expected to be around 1.5 °C between 2030 and 2050, temperatures which may imply severe changes in the worldwide climate, increasing the probability of drought and heavy precipitation in determined regions, along with many increasing risks in fields such as health, food security, water supply, etc. [1]. In order to mitigate these increments and minimize the human impact, stronger policies need to be applied; in addition, worldwide research must provide the technology and resources necessary for the replacement of the contaminating sources by biomaterials and harmless product manufacturing [2,3]. It is at this point that lignocellulosic sources can play a fundamental role as a consequence of their natural origin, ubiquitous production all over the world, minimum carbon footprint and the interesting properties of their main components [3,4,5,6].

Lignocellulosic biomass comprises the skeleton of all the living plants on Earth from their roots, leaves and stalks to their fruits and flowers. Wood is generally divided into two main groups, hardwood and softwood. Whereas hardwood refers to wood coming from angiosperm trees, such as oak, eucalyptus and beech, softwood originates from gymnosperm trees, such as conifers. Both of them are mainly composed of the joined combination of three natural polymers, i.e., cellulose, hemicellulose and lignin, and are currently widely targeted as they comprise promising renewable materials for bioproduct performance and biofuels [6,7,8,9,10,11]. Their content range varies between 40 and 50% of cellulose, 15 and 30% of lignin and 25 and 30% of hemicellulose; nonetheless, these concentrations depend significantly on the type of biomass selected, part and age of the plant, growth conditions [12,13] and part of the cellulose wall [14] (see Table 1).

Cellulose comprises the most abundant biopolymer on Earth, as it is the most important skeletal component of plant cell walls (see Table 1). It is formed by the union of D-glucose units via β-1,4 glycosidic linkages, forming a semicrystalline fibrous structure which can surpass polymerization degrees of 9000 units [17].

On the other hand, hemicellulose does not possess a defined structure, as it consists of a combination of several diverse monomers, i.e., xylose, arabinose, mannose, galactose, rhamnose, glucose, etc. [18], whose concentrations depend on the biomass. Unlike cellulose, it is composed of an amorphous and branched polymer in which monomer units are usually within the range of 500–3000 units [19,20]. The combination of the diverse units usually generates four main hemicellulose structures, i.e., xylan, xyloglucan, galactomannan and galactoglucomannan [21].

**Figure 1:**
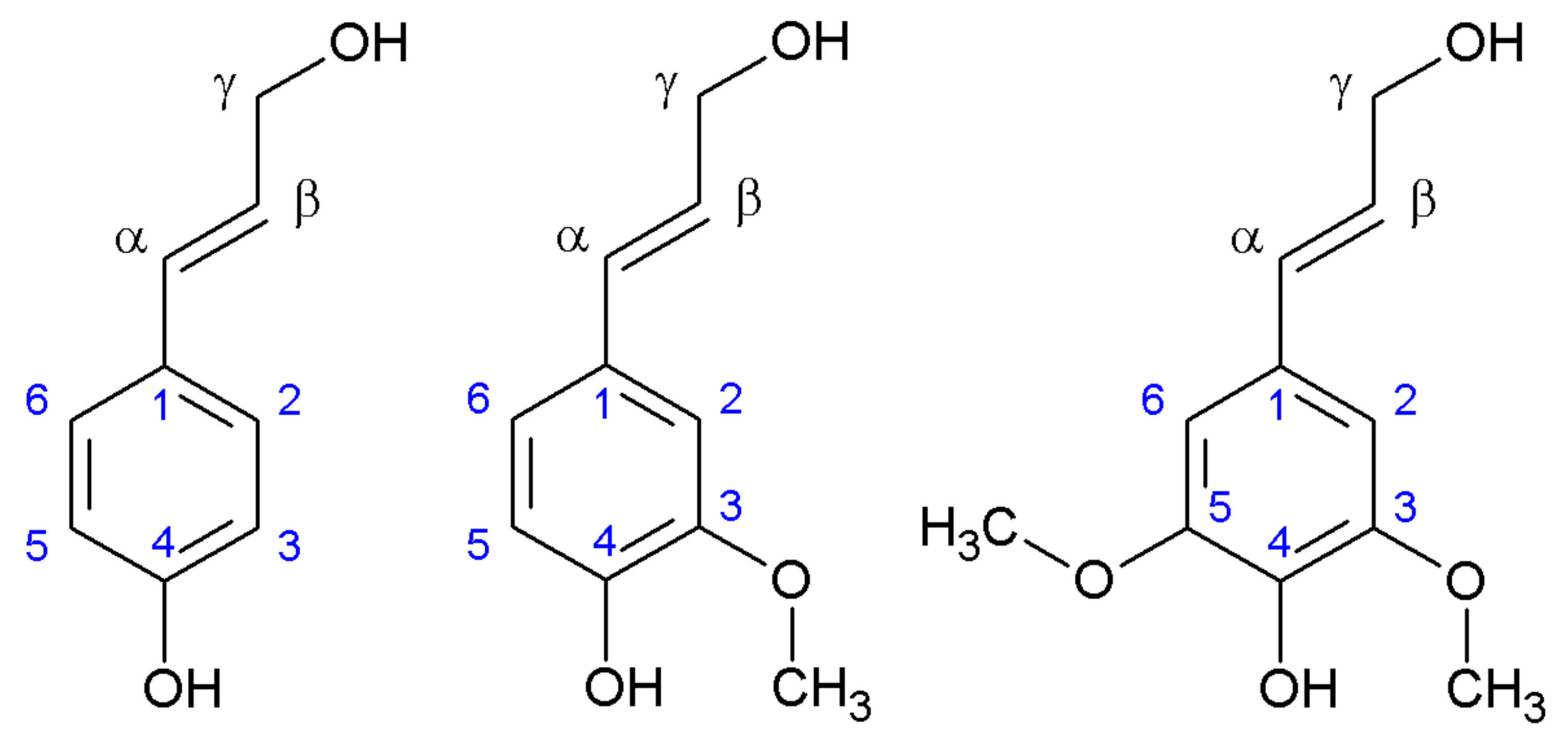
Main monolignol units. p-coumaryl alcohol, coniferyl alcohol and sinapyl alcohol, from left to right.

Lignin consists of a highly entangled biopolymer based on several phenyl propane aromatic units, known as monolignols, together with other aromatic and non-aromatic units. Basically, these monolignols are three, i.e., coniferyl alcohol, sinapyl alcohol and paracoumaryl alcohol, shown in Figure 1.

**Figure 2:**
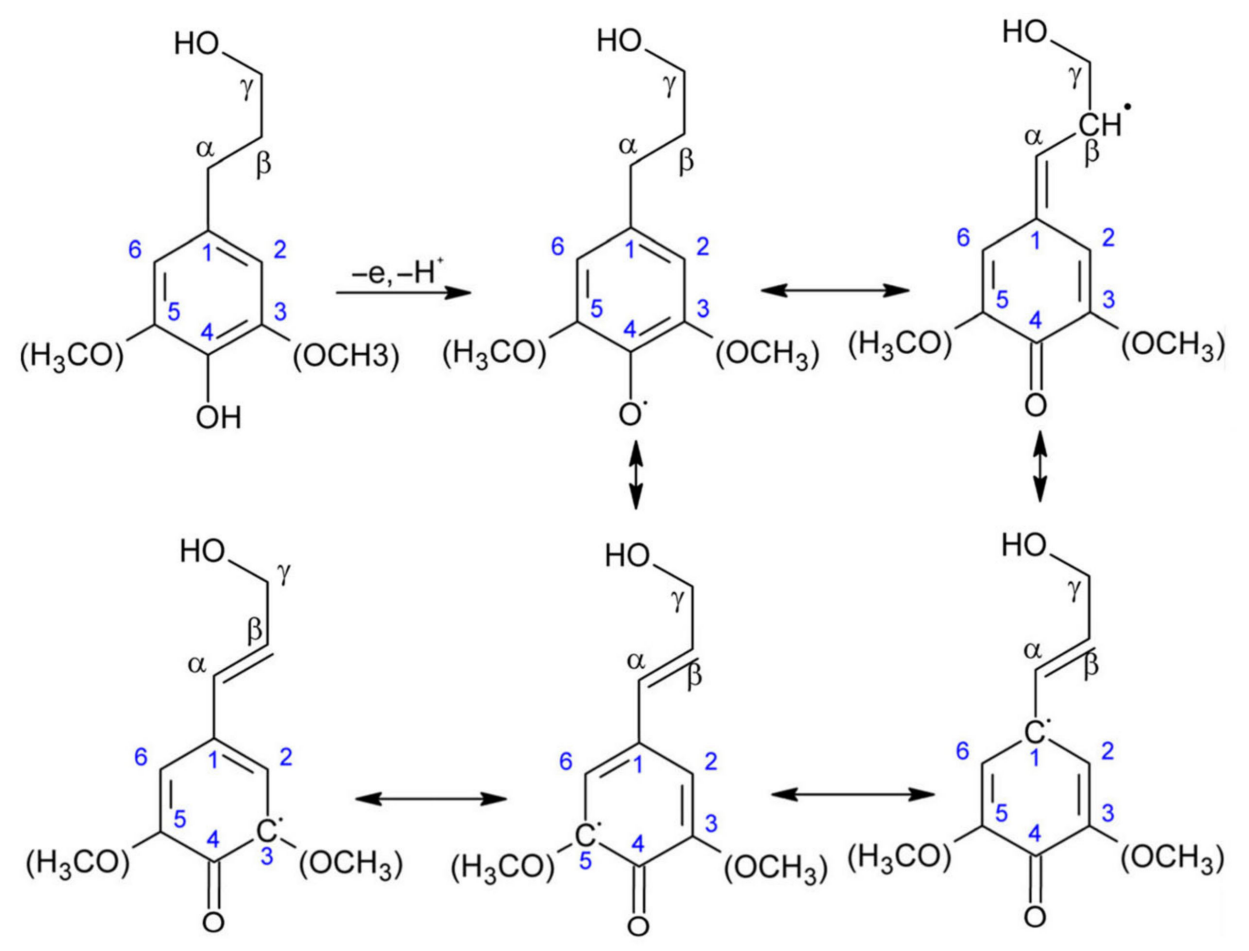
Resonance units of the radical intermediates of the diverse monolignol units during lignin synthesis.

**Figure 3:**
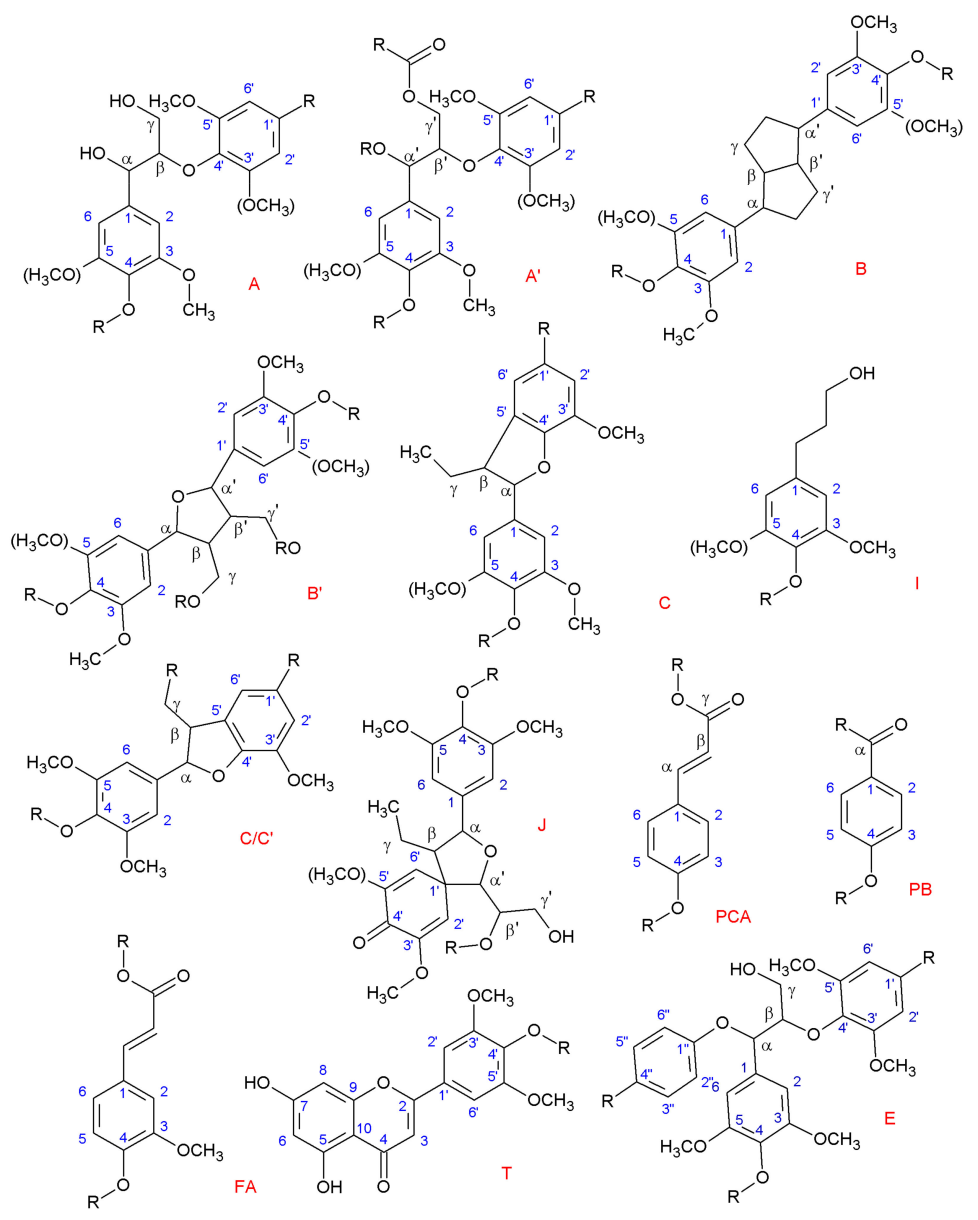
Main lignin structures identified by NMR. (R may indicate both aliphatic and aromatic chains.) (A) β-O-4 alkyl-aryl ethers; (A’) β-O-4 alkyl-aryl ethers with acylated γ’-OH with p-coumaric acid; (B) resinols; (B’) di-c-acylated mono-tetrahydrofuran structure formed by β–β’ coupling and subsequent a-O-a’ bonding (R, acetyl/p-coumaroyl); (C) phenylcoumarans; (I) p-hydroxycinnamyl alcohol end-groups; (C/C’) γ-acetylated phenylcoumaran (R, acetyl) (J) spirodienones (β-1′); (PCA) p-coumarates; (PB) p-hydroxybenzoate; (FA) ferulates; (T) tricin incorporation into the lignin polymer through a G-type β-O-4 linkage; (E) α,β-diaryl ethers (α-O-4/β-O-4).

However, the possibilities of the lignin structure are immense, as just by the combination of those three units, and keeping in mind the different resonance structures that can be generated for further reaction (see Figure 2), a complex and wide range of bonding alternatives is available, making lignin structure extremely difficult to predict [22]. Nonetheless, thanks to powerful nuclear magnetic resonance (NMR) techniques, such as the two-dimensional NMR (2D-NMR) or ^13^C NMR, some of the principal sequences have been elucidated, which are shown in Figure 3 [23,24,25,26]. Further information and extended work in 2D-NMR structural information of lignin can be seen elsewhere [27]. ^13^C NMR detailed information for lignin structure can also be found in the literature [28,29]. 

2D-NMR technique also lets the different percentage of structural units be estimated [23,24,26,30,31]. Thus, the quantification of these interunit linkages has been widely reported, and as can be observed, β-O-4 is outlined as the most prevalent bond type (see Table 2) [32].

All these three materials together produce a highly developed 3D network, which has made the separation of these biopolymers an extremely interesting topic, due to its inherent relationship with the papermaking industry. It has been the case that meticulous research has been carried out in order to improve this separation and hence decrease energy costs [34]. The most followed procedure, the Kraft process, considers both hemicellulose and lignin as low-value byproducts, both being generally used as low-value fuels to recover part of the energy consumed during the process. 

Nonetheless, the full and/or alternative utilisation of the three components would lead to a much better economic performance, which is the basis of the biorefinery concept. An example of how a paper industry could lead to complete exploitation of the raw biomass can be found elsewhere [35].

## 2. Pretreatments of Lignocellulosic Biomass

**Scheme 1:**
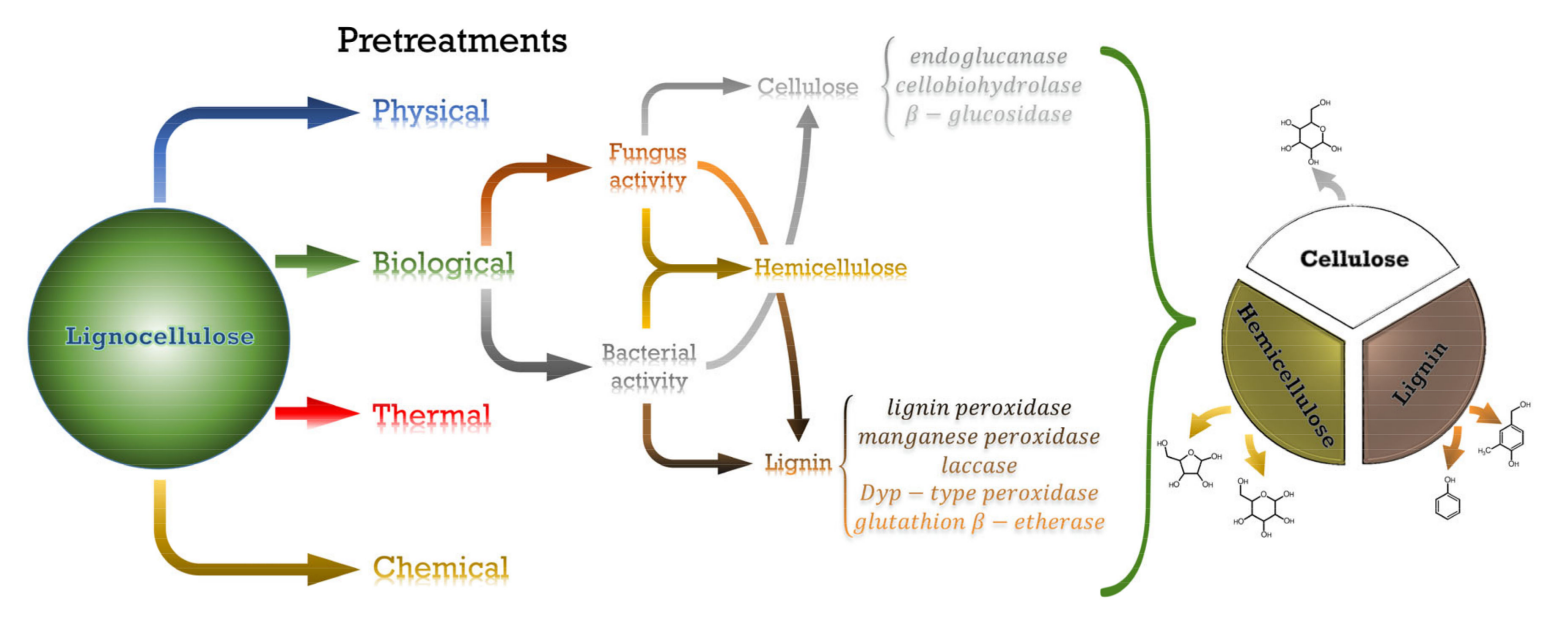
Main pretreatments of lignocellulose and products obtained, with special attention to the enzymes involved in both fungi and bacterial biological processes of cellulose and lignin degradation.

Lignocellulosic biomass has naturally developed protection against enzymatic and pathogen activity through a great entangled network in which the three biopolymers are covalent- or hydrogen-bonded, conforming to a great resilient structure [36], which now plays against technological needs when the disruption of the plant cell wall is aimed at [37]. In order to ease the proper separation among the different lignocellulosic biopolymers, a wide range of pretreatments is available [38]. Furthermore, when specific purposes that require determined properties are aimed at, the application of pretreatments of a different nature becomes necessary. These pretreatments have been classified into four categories: physical, thermal, chemical and biological pretreatments [37,39]. Nevertheless, within a determined process, changes in parameter conditions may anyway lead to diverse structural changes [40]. A descriptive scheme summarizing the main content of this section has been included as Scheme 1. 

### 2.1. Physical Pretreatments

The physical pretreatments include those processes which aim to disrupt the plant cell wall mechanically, reducing the particle size and exposing a higher surface for later purposes. Frequently, these are often preliminary stages after which other pretreatments may be applied. Diverse ball milling procedures are some of the most researched examples [4,37].

### 2.2. Thermal Pretreatments

Among the most used thermal pretreatments, examples such as steam explosion and hydrothermolysis (also known as autohydrolysis) can be found. By taking advantage of water at high temperatures, hemicellulose has been almost completely recovered [4], while lignin and cellulose have been mildly modified, making them more accessible for further treatments. However, the energy requirements in order to implement this technique industrially still remain too high [39].

### 2.3. Chemical Pretreatments

With the chemical pretreatments, the solubilisation of the biopolymers which comprise the lignocellulosic biomass into different solvents is sought. The vast majority of the lignocellulosic separation processes are chemically based, and can be classified into acidic, alkaline and oxidative pretreatments.

The three of them mainly affect both hemicellulose and lignin, leading to great solubilisation of these two biopolymers and making cellulose available to a greater extent for subsequent treatments [37]. For some authors, steam explosion and hydrothermolysis are considered acidic pretreatments as a consequence of the acidic characteristics of water at high temperatures [37]. Detailed information about the diverse chemical pretreatment methods can be found elsewhere [4,36,37,38,39].

### 2.4. Biological Pretreatments

In opposition to the other types of pretreatments, biological procedures have the advantages of being low-energy and low-chemical-consuming processes, eco-friendly and without the formation of inhibitors such as aldehydes, furfurals and phenolics [36,37,39]. Carried out through the action of either fungi or bacteria, the degradation capacity relies on the production of a great variety of enzymes, which are able to digest the three biopolymer types. The main parameters affecting biological pretreatments are the incubation temperature and time, moisture content, pH, aeration, inoculum concentration, particle size and type of microorganism. Moreover, the biomass type also plays a fundamental role [41]. Hereby, the correct scalability of the pretreatment to an industrial process lies in the correct selection of the above-mentioned parameters [39,41]. Although the varying biopolymer degradation by both fungi and bacteria will be discussed in the following sections separately, it is worth noticing that the synergistic activity of the different enzymes is responsible for the complete degradation carried out by the microorganisms [42].

#### 2.4.1. Biodegradation by Fungus Activity

Fungi are able to secrete a wide range of enzymes with the capacity to degrade the three biopolymer types of plant cell walls. Within that range, enzymes can be divided into two types: hydrolytic, able to degrade both cellulose and hemicellulose, and oxidative, mainly responsible for lignin degradation [43]. Fungi can also be divided into three diverse groups, soft-, white- and brown-rot fungi, a classification that is based on the degradation mechanism pattern for each lignocellulose biopolymer [44,45]. Therefore, the white-rot fungi are known to successfully degrade the three lignocellulosic biopolymers. The brown-rot fungi attack is mainly centred on the holocellulose instead, whilst action in lignin is only limited. Finally, the soft-rot fungi demonstrate no effect on lignin [45].

##### Cellulose Biodegradation by Fungus Activity

Regarding cellulose biodegradation, those enzymes with the ability to digest cellulose are called cellulases. Three are the main cellulase groups generated by fungi, i.e., endoglucanases, cellobiohydrolases and β-glucosidases [44]. Endoglucanases are known to randomly attack the amorphous region, opening suitable locations for the subsequent hydrolisation of crystalline structures by cellobiohydrolases and β-glucosidases, which are known to act synergistically [44,45]. 

##### Hemicellulose Biodegradation by Fungus Activity

As a consequence of the greater heterogeneity of this biopolymer in comparison to cellulose, a higher number of enzymes is necessary in order to properly degrade the biopolymer. Each one of the typical monomers that compose hemicellulose, i.e., xylose, mannose, galactose, arabinose, etc., has its own range of enzymes suitable for its proper degradation and transformation [46]. Generally, at least two types of enzymes may be present for each monomer, a first one responsible for the transformation of the hemicellulose chain into oligosaccharides and a second one which acts for the ulterior degradation to the monomers and acetic acid [44].

##### Lignin Biodegradation by Fungus Activity

The most common oxidative enzymes produced by fungi are phenol oxidases, from which lignin and manganese peroxidases and laccases have been more deeply studied. The first ones are responsible for the degradation of the non-phenolic units, whereas the second ones are known to attack both phenolic and non-phenolic units. Laccases, however, only degrade phenolics and other electron-rich groups. The different routes for lignin degradation by using both laccases and peroxidases have been described elsewhere [47]. The lignin breakdown by those enzymes could lead to the production of important aromatic chemicals. For instance, a wide range of aromatic carboxylic acids and acyclic 2,4-hexadiene-1,6-dioic acids were found when spruce-based lignin was degraded by *Phanerochaete chrysosporium*. In this particular case, lignin degradation is occurring by C_α_–C_β_ oxidative cleavage as suggested by the benzoic-acid derivative nature of the compounds obtained [48]. Detailed information about the scheme and further information about fungi-based lignocellulosic degradation can be found elsewhere [44,47,49].

#### 2.4.2. Biodegradation by Bacterial Activity

By producing different types of enzymes, bacteria are also able to degrade lignocellulosic biomass in the same way that fungi do. However, as bacteria generally do not produce lignanases, the aromatic biopolymer constitutes a barrier for many of these bacteria. Such is the interest in the bacterial ability to degrade lignocellulose that some genetic modifications have been carried out in order to improve the degradation capacity [50] or target some specific degradation products [51,52]. The search for bacterial activity related to lignocellulosic degradation has traditionally been performed in the animal gastrointestinal system, but interesting microorganisms have also been found in landfill sites lately [53,54]. 

##### Cellulose Biodegradation by Bacterial Activity

Many specific bacteria have shown the ability to degrade cellulose, such as those coming from the genera *Sporocytophaga, Trichonympha, Cellulomonas Erwinia, Clostridium, Acetivibrio, Thermobifida, Mucilaginibacter, Bacteroides, Streptomyces, Cytophaga, Butyrivibrio, Fibrobacter, Pedobacter, Ruminococcus, Methanobrevibacter, Caldicel-lulosiruptor* and *Clostridium* [21,55,56,57], which come from both aerobic and anaerobic types of bacteria. Generally, likewise, fungi and aerobic bacteria possess the three types of enzymes acting synergistically [57], while the digestion by anaerobic procedures is based on the formation of complexes called cellulosomes (calcium- and-thiol-dependent multicomponent complexes) acting on bacteria’s surface [58,59]. Cellulases have also been divided into families that share a distinctive catalytic core, thus exhibiting a similar reaction mechanism, i.e., either a single substitution with the inversion of the configuration or a double substitution leading to the maintenance of the β-arrangement at the anomeric carbon [60]. Cellulases possess very particular structures, where, along with the usual catalytic domain, many also include domains related to the substrate, cell or cellulosomes binding, the last one leading to the formation of these enzyme-based complexes [60]. These bindings may avoid the elimination of the enzyme from the substrate, conduct hydrolysis to specific domains or facilitate the recovery of the digestion products [60].

Furthermore, often bacteria and microorganisms do not possess the three types, but they act synergistically between them instead. The enzymatic cellulose degradation is affected by both the structural characteristics of the biopolymer (crystallinity, degree of polymerization, etc.) and their own acting enzymes [42,61]. The enzyme-related factors which affect cellulose degradation are enzyme origin, temperature, specific product inhibition, binding to the substrate, activity balance for synergism, specific activity and both enzyme processability and compatibility [42]. 

##### Hemicellulose Biodegradation by Bacterial Activity

Once more, species from both aerobic and anaerobic bacteria were identified as hemicellulose degraders, counting *Ochrobactrum*, *Bacillus*, *Paenibacillus*, *Acinetobacter*, *Thermomonospora*, *Clostridia*, *Streptomyces*, *Cellvibrio* and *Pseudomonas* between the hemicellulase-producer genera [59,62]. In the same way as fungi, complete hemicellulose degradation is accomplished by the synergistic operation of a vast range of enzymes because of the inherent variability of the hemicellulose biopolymer. Only for xylan, a wide range of enzymes has been reported as being mandatory for the degradation completion; these enzymes have been included in Table 3, together with a short explanation of their mode of action [62,63]. 

On the other hand, some xylanases are known not to provoke the breakdown of glycosidic linkages until a proper debranching has been performed. However, those debranching enzymes often require partial hydrolysis before a proper breakage can be obtained. Hence, these findings highlight the intricate complexity of hemicellulose degradation, which, apart from the great numbers of enzymes involved, also requires a careful equilibrium and synergistic operation between the different enzymes [59,63,64].

##### Lignin Biodegradation by Bacterial Activity

Although the ability to degrade lignin is exclusive to a few bacteria genera such as *Streptomyces*, *Rhodococcus*, *Nocardia* or several *Sphingomonas*, *Pseudomonas*, *Enterobacter* and *Actinomyces* species [21,51,65], it has often been reported to be comparable to that shown by well-known lignin-degrader fungi [65]. Furthermore, the difficulties found in the genetic modification together with the low enzymatic yields usually observed in fungi have propelled the interest in the lignin-degrader bacteria [66]. The vast majority of these bacteria have been found within the digestive system of termites and other insects, though some other important species have also been found in soil and decaying vegetation [65,66,67]. 

Despite the enzymology of bacterial lignin digestion not being as developed as deeply as with fungi, some studies have helped to elucidate some of the enzymes which play a significant role in the lignin degradation by bacteria. Thus, four different types of bacterial lignin-degrader enzymes have been found up to this date [66]. Multi-copper oxidase enzymes, also known as laccases, constitute the first one, which have been found in species from the *Streptomyces, Ochrobactrum, Pseudomonas, Paenibacillus* and *Amycolaptosis* genera. Laccases have been found in very different living organisms, in which they play different roles. Hence, they are related to morphogenesis, pathogen-host interaction, stress defence and lignin degradation in fungi, whereas they play an opposite role in plants, where they are responsible for growth by lignin biosynthesis. Functions such as morphogenesis, copper homeostasis and pigmentation have been found in bacteria, whereas they are related to the sclerotisation of the cuticle in insects [54].

Other species from the *Rhodococcus*, *Enterobacter*, *Saccharomonospora*, *Pseudomonas*, *Amycolatopsis* and *Thermobifida* genera have otherwise shown the production of Dyp-type peroxidases [67,68], which form the second type of bacterial lignin-degrader enzymes. Recently, a lignin-oxidising manganese superoxide dismutase enzyme was found in *Sphingobacterium* sp. T2, which establishes the third type of enzymes. The last group is formed by the glutathione-dependent β-etherase enzymes, which are known to act through the β-aryl ether linkage rupture [66]. The visual appearance of the active sites of the different lignin-degrader enzymes can be found elsewhere [66].

The main aromatic species found through the biological pretreatment with lignin-degrader bacteria have been summarised in Table 4, which also includes fungi-related data for this production. 

It is also worth mentioning that such products are not often directly obtained from the lignocellulosic chain; instead, oxidised polymeric intermediates are found. *Streptomyces viridosporus*, *Amycolatopsis* sp. 75iv3 and *Thermobifida fusca* have been shown to produce a water-soluble intermediate described as acid-precipitable poly-phenolic polymeric lignin (APPL) [65,66]. However, the production of either the polymeric intermediates or the direct phenolic-based molecules is based on the cleavage of the interunit linkages that join lignin. Thus, as diverse bonds have been found to form lignin, diverse pathways for lignin breakdown have also been reported, where the β-ketoadipate pathway has been considered the most usual one [54,65,66]. Diverse enzymatic pathways regarding the main interunits that comprise lignin can be found elsewhere [65,69].

As a summary for the pretreatment section, the main pretreatment methods with remarkable characteristics and a description of their advantages/disadvantages have been included in Table 5.

## 3. Lignocellulosic Materials for the Production of Biofuels, Biochemicals and Biomaterials 

**Scheme 2:**
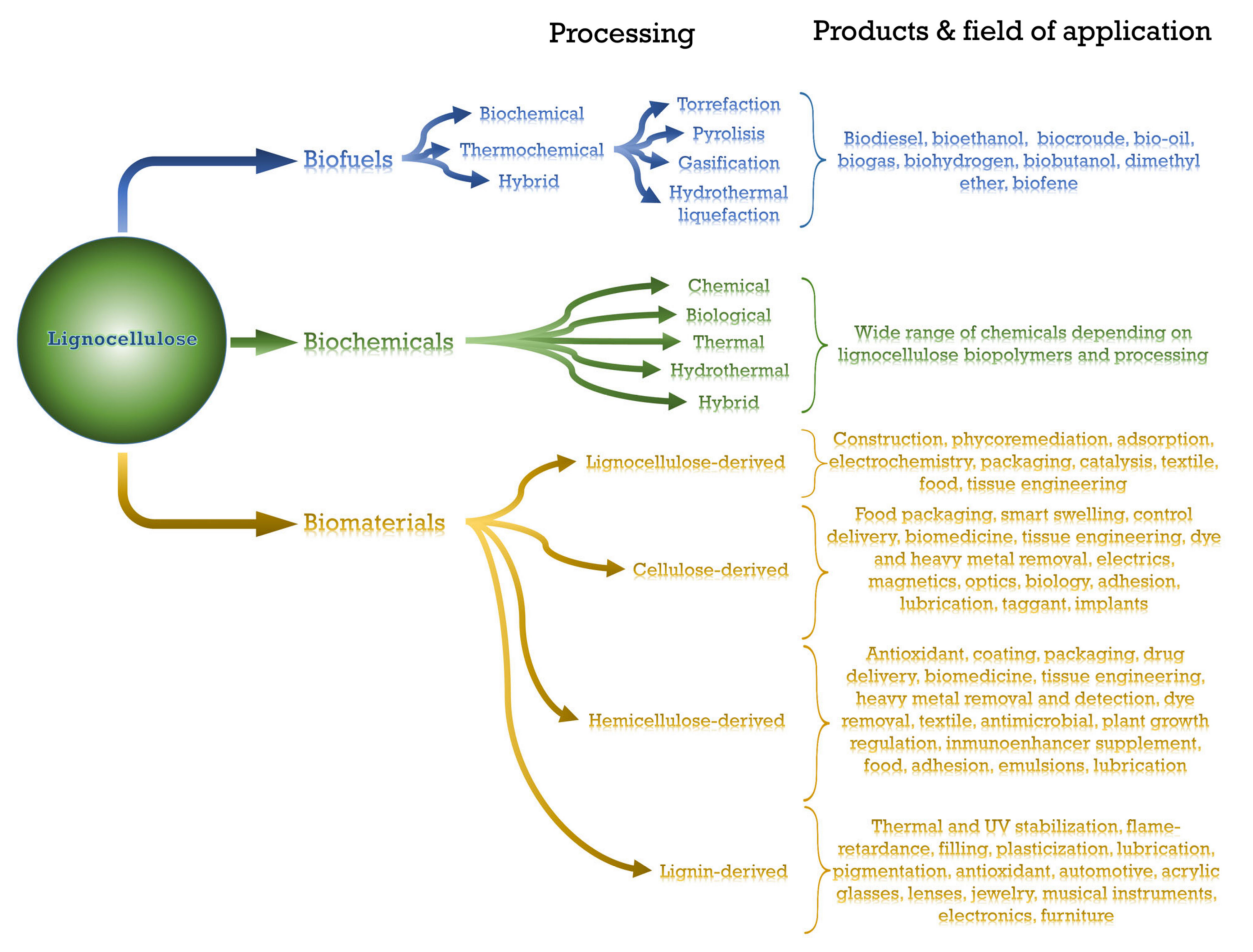
Main procedures, products and fields of application of biofuels, biochemicals and biomaterials derived from lignocellulose.

As previously mentioned, the great availability of a wide range of biopolymers and renewable characteristics has propelled the research into biofuels, biochemicals and biomaterials based on lignocellulosic components [70]. As an overview of this section, the different procedures, products and fields of application of lignocellulose-based biofuels, biochemicals and biomaterials are outlined in Scheme 2.

### 3.1. Biofuels

Compared to petroleum-based fuels, biofuels possess advantages such as renewability, sustainability, availability, biodegradability, safety, neutral greenhouse effects and negligible SO_x_ and reduced NO_x_ gas emissions [71]. Lignocellulose represents the only sustainable, low-cost and scalable eco-friendly option for industrial fuel production [72]. Furthermore, it also represents a great opportunity for increasing the domestic energy production in those countries with large biomass supplies and/or land availability to produce energy crops [73]. The main drawback is found in lignin degradation, as it is considered the more energy-consuming step of the production process, due to the resilience of this biopolymer. Moreover, the cellulose efficiency, enzymatic and biomass costs and composition are other parallel parameters that critically affect the development of suitable technologies [72,73,74]. Furthermore, the obtaining of high-quality biofuels faces other problems, such as many of the most effective solvents for biomass pretreatment being simultaneously incompatible with enzymatic development, whereas those microbes with the highest yields in biofuel production do not often use the sugars present in hydrolysates as substrates [73]. Therefore, as mentioned before and widely discussed, massive attention has been paid to suitable pretreatments for advanced purposes.

Lately, effort has been focused on genome editing technologies as a powerful tool for understanding and developing an integrated system to produce fuels in fast and lower-energy-consuming processes [73]. Thus, some of the inhibitory compounds usually produced in natural plants, such as ferulic acid, can be substantially reduced by genomic editing; nonetheless, negative effects on crop yields, costs and environmental impacts can likewise take place. Another approach that has been substantially developed is the preferential growth of cellulose in detriment of hemicellulose and lignin, as most industrial microbes generally take advantage of hexoses instead of pentoses for biofuel production. Lignin content reduction and composition homogenisation have also been targeted, the latter allowing less complex product mixtures to be generated, thus higher-value molecules are obtained. On the other hand, other strategies have been based on the incorporation of unusual monomers, which potentiates chain elongation or the incorporation of interchangeable linkages, resulting, once more, in higher saccharification yields [73].

Nevertheless, the use of lignocellulosic biomass as biofuel competes with the use as food supply. Hence, a step further can be taken when residual lignocellulosic biomass is considered, as it can be transformed into what is called advanced biofuels, i.e., biofuels which significantly reduce greenhouse emissions simultaneously with the preservation of the common use of landfills, and therefore do not compete with food or feed commerce [70]. In this sense, deep research and many industrial projects which range from aerospace to common fuels, biogas, bioethanol or biodiesel have been accomplished or are being carried out currently [70,72]. In order to obtain those products, three different routes have been targeted, i.e., thermochemical, biochemical and hybrid conversion. An overview of these alternatives can be found elsewhere [70]. Therefore, the action of microorganisms is once again taking the lead as a renewable and environmentally friendly pathway for the conversion of biowaste into biofuels [75].

The thermomechanical route includes a variety of thermal treatments, from relatively low severity to strong processes where high temperatures are applied. Hence, torrefaction, pyrolysis, hydrothermal liquefaction and gasification are found among them. 

The biochemical route relies on the enzymatic digestion of the different biopolymers that comprise lignin, which has been thoroughly described in previous sections, by either fungi or bacteria. Biomass digestion can lead to the production of small sugars, which can be directly used as fuel. However, a suitable separation process is generally needed as a consequence of the huge variety of products involved in lignocellulosic degradation. In addition, by following the biological route, certain compounds that cannot be obtained by chemical routes are produced, opening new areas for advanced biofuels [73].

Often, the products from biochemical routes are further converted by catalytic or thermomechanical processes into higher-added-value fuels. Thus, the hybrid route is accomplished.

The main biofuels that have been studied up to date are summarised in Table 6, including the energy that could be obtained from them. More specific details about raw materials, final characteristics and conversion routes can be seen in the indicated references.

### 3.2. Biochemicals

As a consequence of the great variety of monomeric units and linkages that comprise the three biopolymers, the range of biochemicals that can be obtained from them constitutes an even wider range. These biochemicals critically depend on the original biopolymer, i.e., lignin generally provides outstanding aromatic-based compounds, whereas sugars resulting from the hydrolysis of cellulose and hemicellulose may produce valuable six- and five-carbon-derived products [81]. In addition, the biochemicals obtained are dependent on the processing protocol; therefore, multiple biological and chemical processes, as well as combinations of both, have been studied in order to formulate different chemicals. Special attention is being paid to the bioengineering of these microorganisms; thus, more specific compounds can be targeted. More detailed information about this topic can be found elsewhere [82].

In the case of cellulose and hemicellulose, many different products can be obtained [82,83]. Werpy and Petersen [84] analysed more than 50 compounds, from which they found glycerol, acetic acid, levulinic acid, 3-hydroxybutyrolactone, glutamic acid, malic acid, itaconic acid, aspartic acid, oxalic acid, 3-hydroxy propionic acid, succinic acid, fumaric acid, 2,5-furan dicarboxylic acid, glucaric acid, sorbitol and xylitol/arabinitol to be among the more interesting ones. Most of them can also act as building blocks for the development of fine chemicals and derived compounds. Table 7 depicts the main processes to obtain those species, together with the main derived products that can be obtained from them.

Other important cellulose- and hemicellulose-derived products are 5-hydroxymethyl furfural and furfural, which have been reported as being obtained by hydrothermal carbonisation of those biopolymers, respectively. Both derived furans can also be precursors of many other chemicals, biofuels and pharmaceutical and agrochemical products [81,83,85]. 

A detailed summary including the different chemical structures of both precursors and final products can be found elsewhere [83].

Regarding lignin, as mentioned above, a vast range of aromatic-based chemicals can also be obtained by its decomposition and transformation. However, the success of the suitable formation of chemicals from lignin relies on several main aspects, i.e., lignin fractionation from raw biomass, proper degradation, depolymerisation, transformation into high-value-added compounds and further separation. Depending on depolymerisation conditions, diverse products can be obtained, as shown in Table 8.

The different processes do not only lead to the formation of different species but also the monomer yields depend on the procedure characteristics [86]. 

Similar to cellulose and hemicellulose, several of the compounds obtained can be considered end products, while many others can likewise act as building blocks for possible upgraded compounds [83,86].

### 3.3. Biomaterials

Due to the extensive variety of molecular species obtained from lignocellulosic degradation, the possibilities of derived biomaterials are massive. Werpy and Petersen [84] included in their work an exhaustive review of the biomass components, their primary degradation products, main intermediates and a brief description of derived bioproducts and uses. Hence, lignocellulose derivatives may play a significant role in areas such as general industry, transportation, textiles, packaging and other food vessels, environment, plastic replacers, stationery, house and leisure items, health and hygiene.

However, those biomaterials that make use of the interesting biopolymer network characteristics without further fractionation into derived products are not included among those uses; thus, they will be discussed separately. When considering lignocellulose, frequently research has taken advantage of lignin, cellulose and hemicellulose biopolymers separately; however, the whole biomass has also been considered.

#### 3.3.1. Lignocellulose-Derived Biomaterials

There is an immense range of biosource applications currently, boosted by research possibilities, variety and exceptional properties. Some of them will be discussed further on in this section; nonetheless, a detailed revision of many of these applications is beyond the scope of this work.

As an example, for wheat and barley straw, advanced biomaterials for construction materials, phycoremediation of wastewater, cement properties enhancer and fibres in concrete were reported [87,88,89,90,91,92]. Straws and stalks from other sources were also focused on for board production, being potentially suitable for beaverboard, packing materials, one-use tableware or seeding devices [93,94]. Graphitised lignocellulose extracted from bamboo has also been used lately for electromagnetic wave absorbers [95].

One of the main applications that have been developed is the use of biomass as an adsorbent. In this sense, Rocha et al. [96] studied the adsorption of metal ions such as Cu(II), Zn(II), Hg(II) and Cd(II) on rice-straw-derived solutions. By the formation of biochar from rice straw, other metal ions such as Pb and Zn can also be adsorbed [97], as well as nitrogen and phosphorous [98]. Abdel-Aal et al. [99] reported the ability of rice straw in the treatment of wastewater including several commercial dyes instead. Likewise, banana peel- and palm-flower-waste-based derived products were also able to remove methylene blue and malachite green dyes from polluted solutions [100,101]. Other studies obtained proper adsorbents from residual products such as orange peel and sugarcane bagasse [102]. A detailed review of this matter has been recently published [103].

On the other hand, electrocatalytic activity was also studied on biomass-derived materials. Thus, Castro-Gutiérrez et al. [104] produced tannin-derived carbon materials, while Liu et al. [105] created soybean straw-based Fe-N co-doped porous carbons, both exhibiting excellent properties in electrochemical applications. Ma et al. [106] demonstrated that cornstalks and pomelos skins efficiently act as carbon sources for the construction of cathode catalysts for microbial fuel cells. Another biosource, watermelon, was used by Wu et al. [107] to create hydrogels and aerogels with electrochemical applications. Other biomass sources suitable for electrochemical applications are sawdust or grasses [108]. 

Composites containing residual lignocellulosic biosources have also been targeted. In those, lignocellulose acts as a reinforcing filler and avoids problems such as lack of flexibility or respiratory illnesses [109]. Bugatti et al. [110] showed how tomato peels could form proper composites with halloysite nanotubes for packaging applications. Ita-Nagy et al. [111] demonstrated that sugarcane bagasse fibres also properly reinforce composite structure. Pinhao and pecan nutshells were also used for reinforced composites preparation. The pinhao-nutshell-based composite exhibited lower water absorption capacity than the petcan-based one, based on the enhanced hydrophobic character of the pinhao-based composite [112]. Fibres from tropical maize and sweet sorghum bagasse were also studied as composite additives [113].

The use of biomass as catalysts or catalyst supports has also been deeply studied, as is the case for soybean and other biomass with high protein content [114]. 

In textile, bamboo fibres can provide comfortability, good dyeing and appealing characteristics. Hemp can also be utilised for textile application, as well as for making sacks and ropes, degumming, etc. [115].

In food, tomato peels have acted as an enhancer for colour and antioxidant properties for yoghurts [116], whereas tomato peel fibres have demonstrated the ability to produce a suitable network for edible gels, with enhanced stability and texture [117].

A singular case can be considered when lignocellulosic biomass acts as a hydrogel precursor, which has led to interesting applications being found in fields such as film formation, high-strength filaments, tissue engineering, among many others [118].

Nonetheless, the number of applications and studies is boosted when the different lignocellulosic biopolymers are considered separately. In the following sections, cellulose-, hemicellulose- and lignin-derived biomaterials have been examined.

#### 3.3.2. Cellulose-Derived Biomaterials

The singular structure of cellulose, together with its possibilities of being modified by chemical reactions or converted into alkyl-derived or nanosystems, has made a great range of biomaterials available currently from this biopolymer.

The high number of hydroxyl groups present in cellulose has attracted research attention to the formation of cellulose-based hydrogels. Even though the cellulose ability to be dissolved in water is limited, the development of many suitable solvents has caused hydrogels with stunning properties to be obtained, with applications such as food packaging, smart swelling, controlled delivery and biomedical applications [119,120,121].

The well-known ability of some bacteria to produce cellulose (bacterial cellulose) has also been leveraged for the performance of hydrogels. In general, good tensile and compressive properties are shown, together with high water-absorption capacity, crystallinity and biocompatibility, which have caused bacterial-cellulose-based hydrogels to be focused on bio-applications such as dental and meniscus implants, or tissue engineering scaffolds [119].

Hydrogels from alkyl-cellulose-derived products have also been analysed, such as hydroxypropyl-, hydroxypropylmethyl-, carboxymethyl- or methyl-cellulose. The alkyl-derived chains introduce regions where physical crosslinking may be dampened; thus, chemical crosslinking has frequently been targeted, which has provided hydrogels with new characteristics, for instance, pH dependence in sorption capacity. These hydrogels have been shown to be valuable in water body elimination through the absorption of water in the stomach [122], dye elimination [123], food and drugs [119].

Nonetheless, the capacities of cellulose-based hydrogels can be further boosted by the combination of other synthetic or natural polymers. Hence, heavy metal elimination or food and tissue engineering applications have been targeted by the combination with chitosan, starch or alginate, respectively [119].

On the other hand, inorganic materials have also been added to the cellulose-based hydrogel structures, with applications in fields such as electricity, magnetics, optics and biology. A summarised insight on the potential applications of cellulose-based hydrogels can be found elsewhere [118].

However, not only hydrogels have been reported as being developed from cellulose. Aerogels, usually obtained by freeze-drying of hydrogels or supercritical drying with CO_2_, have also been produced. In the same way as with hydrogels, cellulose, bacterial cellulose and many derived systems (from alkylated compounds to nanosystems) have been reported to produce aerogels, affecting both the synthesis process and final properties [124].

Aerogels possess characteristics such as very low density (up to 0.5 mg/mL), high specific surface area (up to 975 m^2^/g) and highly porous structures (up to 99.9% porosity), while keeping good mechanical characteristics, which have propelled their use in applications such as shock absorbers, acoustic and thermal insulation, oil absorption, biomedical devices and implants, conductivity enhancers or carriers of metal nanoparticles and oxides [124,125,126]. 

On the other hand, by the combination with oily systems, oleogels have also been prepared. Once more, prepared through the use of cellulose and cellulose-derived materials, a vast range of products has been documented. In the food industry, it is ethyl cellulose which has attracted the most attention due to its appealing thickening properties, though pristine cellulose, methylcellulose and hydroxypropyl methylcellulose have also been studied [127]. Nonetheless, the rheology-modifier characteristics have been leveraged for its use in a wider range of applications such as binders, films, adhesives, lubricating greases and hot blends [128,129].

The case of lubricating greases remains especially appealing, as, regardless of the extensive work found in literature, the industry continues to employ almost entirely petroleum-based products and uses lithium- and other metal-based soaps as thickening agents. Hence, studies that explore the use of cellulose pulp as a thickener can be found [130,131,132], but also pristine cellulose [133,134] and cellulose derivatives [133,134,135]. A wide range of these systems has been demonstrated to impart suitable rheological and excellent tribological properties, along with appropriate mechanical stability comparable to lithium-based lubricating grease benchmarks.

As already introduced in this section, the formation of nanostructures from cellulose is also a very appealing approach for the development of potential systems with a broad range of applications. By the formation of nanofibres, excellent properties of water-based hydrogels have been shown, as only a very low concentration is needed in order to obtain good rheological properties. Furthermore, the formation of films and nanocomposites has also been extensively reported. Thus, applications such as reinforcing agent for paper, greaseproof paper, thermosetting resins, strengthened composites, obesity-precautionary thickener, suspension stabiliser, sanitary products, wound dressing, coatings, etc. can be found [136,137].

The formation of cellulose nanocrystals has also been extensively studied, with the majority of applications based on the formation of composites. Nonetheless, the suspensions containing these cellulose nanocrystals have shown nematic chirality, which boosts their applications in fields such as NMR spectroscopy and optical taggants [136,138].

In addition, not only mechanical and chemical processes have been documented to successfully produce cellulose nanostructures. Instead, some bacteria have also exhibited the possibility of directly obtaining cellulose nanostructures with the only presence of hydroxyl moieties as functional groups. The high yield for a biological process (up to 40%) and unique structure have propelled its use in fields such as regenerative medicine, wound healing, implants, membranes, films and barrier layers [136].

#### 3.3.3. Hemicellulose-Derived Biomaterials

Even though hemicellulose does not possess the significance and properties of cellulose and cellulose derivatives, there are also many studies that take advantage of hemicellulose structure to produce interesting biomaterials. Due to the fact that hemicellulose is not formed by a single type of biopolymer, the possibilities are again raised. The main target of hemicellulose-based biomaterials has been based on the production of antioxidant agents, hydrogels and films for uses such as coatings, packaging or biomedicine; nonetheless, some other less common uses have also been studied.

Hydrogels have been mainly centred on drug delivery [139,140,141], tissue engineering and environmental protection, and have demonstrated pH, ionic strength, media composition and organic-solvent-dependent behaviour. Thus, the removal of heavy metals such as Ni (II), Cu(II), Pb (II), Cd(II), Pd(II) and Zn(II) or sulfadimidine has been successfully achieved by these hydrogels [142,143,144]. On the contrary, good adhesion in tissues like liver has also been documented, in which they may play a good replacement role in detriment to more expensive and less available tissue and organ transplants [145]. In drug delivery, xylan- and galactomannan-based microcapsules have been shown to be excellent colon-specific carriers [140,146,147], while xyloglucan mucoadhesive and surface tailoring properties have led to the development of many specific studies [141]. Galactomannans have also been shown to be useful for drug delivery by the formation of an aerogel structure [148]. 

Hemicellulose-based films were also produced, whose significance is based on their outstanding properties against oxygen permeability, thus conforming to a great replacement for oxygen-sensitive food packaging. Xylan, arabinoxylan, glucomannan and galactoglucomannan, alone, modified or by combination with other biopolymers, are some of the biopolymers from hemicellulose which have shown suitable properties for film production [143,149,150]. 

The same oxygen permeability provides hemicellulose with interesting antioxidant and antimoisture properties, which make them good replacements as coatings for food packaging. 

Between the less common uses, there is still a vast range to be found. Peng et al. [151] demonstrated that hemicellulose acts as a stabiliser for the formation of silver nanoparticles. Jiang et al. [152], instead, used hemicellulose for the synthesis of quantum dots to detect Ag(I) and L-Cysteyne in aqueous solutions. Farhat et al. [153] were able to produce hemicellulose from bleached hardwood pulp and switchgrass, which, crosslinked with zirconium, exhibited excellent adhesive properties. Xylan has been analysed by Ebringerova [154], who evaluated other potential applications such as textile printing, antimicrobial additive, plant growth regulator, inmunoenhancing supplement, additives and thickening agent in food. On the other hand, xyloglucan has also been reported as a texture enhancer, binder, dye absorption, emulsion stabilizer, syneresis control and food additive [155]. Some of these applications are shared by galactomannan-derived products, which have been shown to act as binders, texture modifiers, emulsifiers, lubricators or stabilisers, mainly in the food industry [156]. Galactoglucomannan has also been studied as a food additive because of its prebiotic properties [157].

#### 3.3.4. Lignin-Derived Biomaterials

The unique characteristics of lignin have propelled both research and industry to focus on this biosource acting as an additive for a wide range of polymers, such as polypropylene, polystyrene, polyethylene, polyamide, PVC, poly(vinyl alcohol), among other bio and synthetic polymers [158]. Thus, thermal and UV stabiliser, flame-retardant, reinforcing filler, plasticiser, lubricant, colour-adding pigment and antioxidant are among the most significant properties that lignin incorporation can enhance or modify [159], producing interesting biomaterials that can be applied in fields such as thermoplastic, thermoset, bioplastic and rubber composites, aerogels, carbon fibres and foams [158].

For instance, lignin addition in polyethylene and polystyrene matrices up to 20% has been shown not to modify processability whilst significantly improving resistance against photodegradation [160]. In another study, Mishra et al. [161] demonstrated that lignin provides stronger resistance to UV in PVC films acting as UV absorber. Yang et al. [162] also showed that poly(methyl methacrylate) thermal and mechanical properties are enhanced by the lignin addition against UV light. Therefore, applications in fields such as automotive, acrylic glasses, vehicles and lenses have been targeted. On the other hand, different lignin fraction response to UV light as a function of the solvent utilised has also been studied, showing UV resistance to be dependent on the lignin extraction method [163,164]. In Sheng et al. [164], the phenolic hydroxyl group content of several residual lignins turned out to be crucial to their performance as antioxidants. 

Thermal resistance is frequently another weak point in conventional polymers and composites; thus, lignin addition as enhancer of the heat resistance has been a deeply studied topic. Canetti et al. [165] demonstrated that lignin addition from 5 to 10% in polypropylene blends successfully improves the thermal resistance. Working on natural rubber modification, Gregorová et al. [166] showed that the lignin addition also increased the thermo-oxidative long-term resistance. On the contrary, Tavares et al. [167] reported that a simple 1% lignin addition reinforces the poly(butylene adipate-co-terephthalate) matrix. On the other hand, Lisperguer et al. [168] demonstrated that lignin addition to recycled polystyrene could provide similar characteristics to the native polymer. Such is the interest in the improvement of the thermal properties by lignin addition of blends, composites and copolymers that Sen et al. [169] wrote an extensive review where lignin modification by different processes and the thermal response of the products obtained were evaluated.

Flame prevention is another powerful characteristic that lignin can provide to a biomaterial. Thus, De Chirico et al. [170] worked with lignin and various derivatives, which were able to provide the polypropylene matrix with enhanced combustion time and char yields and reduced both heat liberation and mass loss rate while saving mechanical properties. The flame retardant properties of polylactic-acid-based biopolymers were likewise improved by the use of lignin nanoparticles functionalised with diethyl (2-(triethoxysilyl) ethyl) phosphonate [171]. Another renewable polymer, polybutylene succinate, was also successfully treated with nitrogen-and-phosphorous-doped lignin systems, improving both heat release rate and total heat release in around 30%, properties that could lead this biopolymer to be applied in wider fields [172]. Recently, some reviews concerning the flame retardant possibilities of lignin and its derivatives, as well as their future prospects, have been reported [173,174].

Lignin acting as reinforcing filler has also been extensively documented. Therefore, Ikeda et al. [175] showed that lignin potentially improves tensile stress and storage moduli of natural rubber, while reducing the dissipative loss. The tensile and flexural modulus of polylatic acid, poly(3-hydroxybutyrate) and thermoplastic elastomers after lignin addition were also raised [176]. Mechanical properties were also generally improved when lignin nanoparticle-poly (diallyldimethylammonium chloride) complexes were added to natural rubber [177]. In a study developed by Rozman et al. [178], coconut fibre and polypropylene were mixed using lignin as compatibiliser. The mixtures exhibited better flexural properties when lignin was included. On the other hand, Tanjung et al. [179] demonstrated that lignin addition to polypropylene/chitosan composites successfully increased tensile strength, elongation at break, Young’s modulus and impact strength. Extensive reports have been documented regarding lignin acting as filler for bioplastics, thermoplastic and thermoset composites [158]. Regarding bioplastics, Yang et al. [180] revised the most recent literature in lignin-reinforced bioplastics made of cellulose, protein, starch, polylatic acid and poly-hydroxybutyrate. Recently, an extensive review aiming to compile the latest advances in lignin-reinforcing properties in the rubber industry has also been published [181].

As mentioned above, lignin can likewise act as plasticiser, which has been achieved frequently through structural modifications. Therefore, lignosulfonate has been used as the usual plasticiser for concrete. Through other modifications like alkylation, the plasticising effect of lignin has also been achieved [169]. Working with PVC and different molecular weight lignin fractions, those with the lowest ones were reported to act as plasticisers by Yue et al. [182]. On the other hand, a novel process consisting of an alkali-O_2_ oxidation technology for lignin revalorisation has recently been addressed for plasticisation, (LigniOx), which was included in the annual report of the top 20 innovative bio-based products form the European Commission [183]. Moreover, by further modifications, superplasticisers have been targeted, which could replicate the performance of well-known commercial naphthalene plasticisers [184].

The presence of hydroxyl groups in lignin has provided it with the possibility of establishing H-bonds, which has been leveraged for its use as an additive for lubricants. Hereby, lignin has been added to many different base oils, in which the H-bonds formed have proved to generally decrease both wear and friction. Mu et al. [185] developed fully bio-based lubricants by utilising lignin and ionic liquids, demonstrating outstanding tribological and anticorrosive properties on both aluminium and iron surfaces. Working with polyethylene glycol as the base oil in a more recent study, Mu et al. [186] compared lignins from different origins and extraction processes as additives, highlighting hydrogen bonding and molecular weight as crucial factors on thermal and lubricating properties. Up to 93.8% wear reduction was reported by the lignin incorporation. In another study, Hua et al. [187] created lignin-based green lubricants with excellent properties in diamond-like carbon-steel contact compared to commercial lubricants, showing lignin-based lubricants to be useful on surfaces with different characteristics. Cortés-Triviño et al. [188] tested a lignin-enriched residue coming from sugarcane bioethanol production to further valorise it by mixing with castor oil epoxidised at different degrees, showing the possibility to tune the rheological and lubricant properties thereof of the mentioned mixtures. The production of nanofibres from lignin by using the electrospinning technique has likewise permitted the use of these materials as oil structuring [189]. In addition, not only liquid lubricants but also semi-solid ones, i.e., lubricating greases, have been developed by using lignin [190,191].

Even though lignin is almost colourless in wood, its separation from cellulose and hemicellulose finally turns it into a dark brown colour. For this reason, Balasubramanian et al. [192] used lignin as brown pigment and tested it in leather, where, apart from dyeing the surface properly, it exhibited compatibilisation with common products for leather finishing. On the other hand, Araújo et al. [193] used lignin nanoparticles to encapsulate blue pigments, which increased both solubilisation and stabilisation, making them more suitable for industrial applications. 

Within lignin roles in plants resides the antioxidant capacity as a consequence of its aromatic structure, which has likewise been used for the incorporation of antioxidant properties in biomaterials production, with applications in cosmetics, healthcare, agricultural products and pharmaceuticals [194,195]. Once more, the varied lignin characteristics depending on both the origin and extraction method have allowed adjustable antioxidant properties to be obtained [194,196,197]. For instance, Li et al. [198] used different solvents (ether, ethyl acetate, methanol, acetone and dioxane/water) in order to study the antioxidant properties of the different fractions obtained. The results showed that the higher the dissolving ability of the chemicals, the lower the antioxidant capacity of the resulting lignin fraction. Instead, Ma et al. [199] investigated the antioxidant activity as a function of pH in the lignin extraction method. High pHs led to low lignin content and low phenolic content, which exhibited low antioxidant activity. On the contrary, low pHs turned into lower molecular weight lignin fractions with high phenolic hydroxyl content, showing excellent antioxidant properties and highlighting the crucial effect of both molecular weight and phenolic hydroxyl group content in antioxidant properties. 

Industrially, lignin has been used as a dispersant, binder or chelator, as a substitute of phenolics powder resins, in polyurethane foams and epoxy resins or as biodispersant, among others. Thus, some North American companies have implemented lignin addition in products such as automotive brake pads and moulds and oriented strand boards. The company Bioconsult Gesellschaft fuer Biotechnologie GmbH has developed a lignin-based system able to control the microbial growth in industrial wastewater ambits [200]. The company Nippon Paper Group has released diverse lignin-based biomaterials, SAN X^®^, VANILLEX^®^, PEARLLEX^®^, which can act as mentioned above according to their characteristics [201]. More concretely, the company TECNARO has been able to use lignin in thermoplastics with applications in fields such as jewellery, construction, musical instruments, electronics, furniture, etc. The Prisma Renewable Composites Company developed a system named evolUTIATM, able to create lignins with similar characteristics from different plant sources, which has let them use lignin in biomaterials such as plastics, elastomers and carbon fibres. Their first product is BioLANTM, which has been declared as a substitute for ABS with enhanced mechanical and UV-resistant properties.

## 4. Lignocellulose-Based Polyurethanes

Due to the ubiquitous production, environmentally friendly character and interesting properties of either lignocellulosic sources as a whole or sources separated into their main components, extensive research has been devoted to the formation of polyurethanes based on lignocellulosic materials [202]. For instance, the addition of wheat straw in polyethylene-glycol-based PU foams was shown to provide both better thermal resistance and compressive strength compared to those systems only based on polyethylene glycol. Moreover, biodegradability was likewise enhanced [203]. Wheat straw was once more used in combination with castor oil to produce suitable PU coatings for controlled-release fertiliser. Apart from the great controlled-release characteristics, the PUs exhibited good degradability and high density [204]. On the other hand, barley-straw-derived foams for insulation were also produced, which demonstrated water intakes up to 986% after 48 h, better than those obtained with synthetic PUs [205]. PU foams were also considered by Ertas et al. [206] but were obtained from *Eucalyptus camaldulensis* and *Pinus sylvestris* instead. Other biosources considered for the production of rigid foams were cotton stalk, pine bark and apricot stone [207,208]. Moreover, polyurethane films were successfully elaborated by employing lignocellulose materials such as wheat starch by the reaction with isophorone diisocyanate, which exhibit suitable mechanical properties to replace petrochemical substitutes [209]. Nonetheless, the range of available PU for different applications has been greatly expanded by the use of cellulose, hemicellulose and lignin separately.

### 4.1. Cellulose-Based Polyurethanes

Cellulose-based polyurethanes have been produced by either taking advantage of the biopolymer structure and hydroxyl functional groups or by the cleavage and modification of the structure in order to obtain intermediate products for PU formation. The last one is the case, for instance, for isosorbide and 2,3-butanediol. Both derived from glucose, Calvo-Correas et al. [210] demonstrated they can act as copolymers in PU synthesis for film formulations, which can compete with non-renewable ones. On the other hand, Wei et al. [211] elaborated a synthetic route to produce adipic acid from cellulose, from which Nylon 66 or PUs can be obtained. Another approach was the transformation of cellulose into what is called cellulose-based ionic liquids. These systems were transformed into PUs from which excellent membranes were produced, able to adequately separate CO_2_/CH_4_ from natural gas sidestreams [212].

Cellulose has been frequently employed as Pickering emulsifying agent, from which porous monoliths can be formed. However, poor mechanical properties have been generally observed. Nonetheless, by the chemical interaction with diisocyanates, proper robustness can be obtained. Moreover, the system provides suitable and fast absorption capacity and tunable wettability from hydrophilicity/oleophilicity to hydrophobicity/oleophilicity [213]. Cellulose has also been used as grafting copolymer, which has led to interesting shape-memory products for smart applications [214]. Cellulose from industrial furniture waste has also been tested to absorb dye within a PU foam matrix. For the three dyes utilised, Methylene blue, Procion yellow and Procion red, the kinetic studies suggested a pseudo-second-order absorption model. The maximum removal values were around 70, 90 and 80 wt.%, respectively [215].

Using cellulose fibres, the reinforcement of polyurethane composites has been targeted [216,217]. Hadjad et al. [218] incorporated up to 30 wt.% of cellulose fibres and studied the conductivity and capacitance alterations of the composites. Up to 10 wt.% concentration, the main electrical characteristics were unaffected. However, cellulose can also be used as a chain extender due to the high molecular weight usually reported; thus, Ikhwan et al. [219] developed cellulose fibres and polyethylene-glycol-based PUs, whose relative concentration demonstrated a strong influence on thermal properties. In order to provide PU composites with superior performance, nanofibres have been utilised. The use of nanofibres has provided a fast response (less than 1 min) in shape recovery, which propels the potential use of these nanocomposites for biomedical applications [220]. The improvement of properties by using cellulose nanofibers was also observed for flame retardant applications. Therefore, by assembling with anionic vermiculite, outstanding transparency, resistance to oxygen pass and record fire resistance characteristics have been reported [221]. On the other hand, aerogels with excellent flame retardant properties were also obtained by combination with hydroxyapatite [222].

Polyurethanes were also prepared by using bacterial cellulose. In Urbina et al. [223], biocompatible PU nanocomposites were once more reinforced by bacterial cellulose incorporation, displaying outstanding shape memory and mechanical results. Nonetheless, they could not be compared to the use of nanofibers, as 93% recovery took place in around 3 min.

The use of cellulose nanocrystals was also focused on for the reinforcement of PU nanocomposites. An enhancement on both Young modulus and stress at break was usually recorded [224,225]. 

### 4.2. Hemicellulose-Based Polyurethanes

In the same way as has been happening with its glucose-based homonym, research has been devoted to the production of bio-PUs by following two pathways; using the complete hemicellulose structure and derived constituents or dividing it into smaller units, which can be further used as polyols. Hence, following the last approach, xylitol or furfural have been obtained and applied for PU formation by liquefaction or oxypropilation [226]. Along with xylitol, sorbitol was also produced by Robinson et al. [227]. On the other hand, Samavi and Rakshit [228] utilised hemicellulose liquor to produce epoxidised microbial oil, which could be further used as polyol in PU production. A summary of the main processes which can lead to hemicellulose division into polyols has been explained elsewhere [229].

Taking advantage of the hemicellulose and derivatives structure instead, Cheng et al. [230] reported the use of xylan to produce PUs with enhanced thermal stability. On the other hand, arabinoxylan was used to achieve PU films with application in the packaging field [231], or coatings by crosslinking with glutaraldehyde, which exhibited comparable properties to polyvinyl alcohol, becoming a sustainable substitute [232]. Simultaneously, studies using arabinogalactan have helped polyurethane scaffolds to improve cell attachment yields [233,234]. On the contrary, another hemicellulose-constituent biopolymer, galactomannan, has been shown to provide PUs with excellent characteristics for drug release in specific body parts [235]. Nonetheless, the most used hemicellulose-derived biopolymer is glucomannan. Improving mechanical performance, the use of glucomannan in composites and nanocomposites has been thoroughly studied. Its combination with waterborne PUs has also provided excellent mechanical performance and thermal properties due to the strong H-bonding between the PU and glucomannan [236]. A summary of glucomannan-based products with their potential applications and techniques used for characterisation is included herein below as Table 9.

Furthermore, in a study elaborated by Shao et al. [237], no division but the whole hemicellulose extracted from corncob was used to perform PU films. These were compared to films that originated from cellulose, lignin and diverse mixes between the three biopolymers, obtaining for the hemicellulose-based PU films the highest glass transition temperature.

### 4.3. Lignin-Based Polyurethanes

Similar to the previous biopolymers, both the lignin as a whole structure or derivatives and small compounds obtained from it have successfully produced PUs. Thus, the production of ferulic acid and cresol-based monomers from lignin yielded PUs with superior thermal characteristics [238]. In the same way, vanillin-derived systems were also used as polyols for PU formation [239].

Extensive research has been carried out regarding the use of lignin in polyurethane formation due to the availability of hydroxyl groups, which makes it an interesting biopolyol replacer. Moreover, both aromatic and aliphatic hydroxyl groups have been demonstrated to react with diisocyanates [240]; however, some of these groups may be hindered due to steric encumbrance based on the network ordering and self-association [241]. Generally, lignin has been used alone or accompanied by other synthetic or natural polyols, while often it is first modified to enhance overall hydroxyl group value and aliphatic hydroxyl value or improve access to them. In this sense, demethylation stands as a very useful technique, through which lignin causes methyl groups to be replaced by hydrogen, finally obtaining higher hydroxyl values. Another technique extensively used is hydroxyalkylation, which introduces primary and secondary alcohols to the lignin network by combination with different compounds [241]. Another significant process that is intended to enhance hydroxyl value is phenolation. Since diisocyanates also possess a great affinity to amine groups, lignin amination and nitration have likewise been focused on [241,242,243]. Nonetheless, the high molecular weight and structure stand for a too-high viscosity and difficulties with reactivity; thus, depolymerisation is often targeted as well.

In any case, lignin can provide exceptional fire resistance, crosslink density, ultraviolet stability, biodegradability, insulation, compression, antioxidant, thermal and reinforcing properties to PUs [200,238,241,244,245], as well as high availability and low cost, the reason why foams constitute one of the main formulations for lignin-based PUs, where it can act either as a polyol or as a filler. The mechanical performance is usually improved as a consequence of the more entangled generated systems [245]; however, lignin is also known to increase stiffness, and therefore brittleness, due to the aromatic and low flexible structural configuration, which has been usually reported for concentrations larger than 30 wt.% [241]. As a consequence, flexible chains like castor oil, butanediol and polypropylene oxide have been proved valuable for improving this characteristic. Another approach followed has been to depolymerise native lignin to reduce its molecular weight [244]. It is also worth mentioning that different lignin-extraction procedures, origin, biomass source, etc. can likewise lead to lignin with different properties, which extends to derived PUs [240,246,247]. Therefore, new pathways are always being studied, such as aldehyde-assisted fractionation, which provides lignin with enhanced solubility, functionality and purity compared to the well-extended Kraft process [248]. Despite the mentioned disadvantages, many authors have proved that lignin-based foams act similarly or even better than those petroleum-based ones [238,241]. However, the use of lignin in PUs is not limited to foams. Elastomers, adhesives, coatings and more special products are also frequently targeted [241,245]. Regarding coating applications, Griffini et al. [249] utilised non-modified lignin to produce suitable films with enhanced formation ability, adhesion on diverse substrates, tensile properties and hydrophobic behaviour. Nonetheless, other properties like antioxidation, gas impermeability and UV resistance have also been reported for lignin-PU-based coatings [250]. Thus, Xie et al. [251] developed PUs by using different diisocyanates and Ag nanoparticles, which showed excellent thermal and mechanical properties with very high lignin content (more than 40%), while excellent antibacterial properties were provided by the metal addition. On the other hand, Fuqiang and Xiangjiao [252] developed UV-cured lignin PUs with improved hydrophobicity. In a more complex combination, Hu et al. [253] utilised the combined benefits of carbon nanotubes, Fe_3_O_4_ nanoparticles and lignin to develop PUs with excellent performance as electromagnetic shields. In general, weathering performance was also improved by the lignin inclusion into the polymeric matrix [241]. 

To a lesser extent, lignin has also been used to produce composites that can act as tent fabrics [254], potentiometric chemical sensors [255], chromatographic and chemoselective membrane applications [256] and stereolithography 3D printing ink.

### 4.4. Lignocellulose-Based Polyurethane Adhesives

Since PU arrival, great effort has been made for the production of high-performance PU-based adhesives. As can be observed within the landscape of adhesives established by Burchardt [257], PUs plays a very significant role throughout the whole mechanical/adhesion response spectra. Nonetheless, recent advances in political and environmental restrictions are endangering the use of commercial PU adhesives since they are usually formed by the combination of non-renewable polyols and harmful diisocyanates. As already mentioned above, it is in the polyol substitution where biomass can play a significant role; however, other techniques aiming to reach 100% bio-based adhesives are also to be discussed in this section.

Once more, the crosslinking ability of lignocellulose biopolymers, low cost and renewable character have been leveraged in order to produce bio-based PU adhesives. Therefore, liquefied products have demonstrated a great ability to develop a wide range of products [258]. Thus, the liquefaction of two plant species, China fir (*Cunninghamia lanceolata*) and Taiwan acacia (*Acacia confusa*) was carried out to subsequently produce PU adhesives by combining up to three types of poliisocyanates, i.e., Desmodur L (based on toluene diisocyanate and trimethylol propane), Desmodur N (based on hexamethylene diisocyanate) and poly-4,4′-diphenylmethane diisocyanate. The study allowed one to conclude that liquefied Taiwan acacia and Desmodur L exhibit the best adhesion performance [259]. Different sources, such as the Japanese cherry blossom or Sakura (*Prunus cerasus*) [260], kenaf (*Hibiscus cannabinus* L.) [261] or Sugi (*Criptmeria Japonica*) [262] were likewise tested after liquefaction to produce polyurethane adhesives with poly-4,4′-diphenylmethane diisocyanate. Nonetheless, the fractionation of lignocellulose into its main components is much preferred for PU adhesive production and is discussed in the next sections. 

#### 4.4.1. Cellulose-Based Polyurethane Adhesives

Cellulose and its derivatives have also been employed to produce bioadhesives. Thus, cellulose acetate in combination with CO was used by Tenorio-Alfonso et al. in several studies [263,264,265], from which a wide range of concentrations, NCO:OH ratios and diverse diisocyanates were tested, always exhibiting excellent properties, comparable to commercial adhesives. Similar to lignocellulose, liquefaction was also used to formulate adhesives from cellulose pulp; thus, sugar-beet-derived pulp adhesives with up to 87% biosource exhibited good adhesion ability, improved by increasing diisocyanate/biosource ratio. The stability of sugar beet pulp adhesives was better than another biomass also tested [266].

#### 4.4.2. Hemicellulose-Based Polyurethane Adhesives

As previously mentioned, hemicellulose is formed by a combination of different monomers, such as xylose, arabinose, etc. Balcioglu et al. [267] utilised xylose for PU-adhesive formulations, obtaining adequate biocompatible adhesives with strength up to 415 kPa, at 15% xylose concentration. A patent has been registered where xylan inclusion in adhesives reported strengthening of the bonding [268]. Although any hemicellulose-derived polyol can actually be used as a precursor for PU-based adhesives, no such studies have been performed to our knowledge.

#### 4.4.3. Lignin-Based Polyurethane Adhesives

Lignin has also been widely studied over the last few decades for PU adhesion, as demonstrated by the vast number of publications and patents centred on this topic [241,242,256,269,270]. The use of lignin allows one to overcome typical PU-adhesive drawbacks, such as delamination resistance, cohesive failure and gap-filling properties. Moreover, it generally enhances the thermal behaviour of the adhesive [241].

To mention some of the greatest advances reported, lignin in combination with vanillin-derived diisocyanates was able to withstand more than 9 MPa break at failure onto glass surface, while the results for other surfaces (wood, aluminium and steel) were much lower (around 1 MPa). Moreover, the use of diverse lignin sources demonstrated adhesive properties to be tailored [271]. Griffini et al. [249] also exhibited outstanding adhesive properties of lignin-based PUs; however, the maximum strength was found in wood specimens (more than 9 MPa), while glass still exhibited very good results (7.6 MPa), as well as aluminium and steel, showing around 1 MPa. Very good results in wood-wood surface, but also in metal-metal and metal-textile contact were also reported for lignin-and-castor-oil-based adhesives, where the lignin precedence, either from barley or wheat straw, played a crucial role on the adhesive performance. In this work, biological modifications were also targeted, allowing the tailoring of the adhesion values by the careful selection of both the lignin precedence and the *Streptomyces* strain [270]. As hydroxyl groups are the main bonding point to form proper urethane bonds with diisocyanates, other studies have been focused on primarily increasing the hydroxyl group index of lignin by hydroxypropylation or demethylation in order to create a stronger adhesive network [256,272,273]. In this sense, improved tensile strength of more than 200% reference values was obtained by hydroxypropylation [272]. On the other hand, biological modifications with laccase were leveraged by Ibrahim et al. [274], who tested both uninoculated and inoculated lignin with other natural sources such as chitosan and soy protein as well as with polyethyleneimine to produce suitable adhesives. These exhibited close values to petroleum-based ones and withstood hard thermal treatments with no significant performance loss. Regarding systematic studies aiming to elucidate main parameters for adhesion evaluation, Lima-García et al. [275] tested different kinds of lignins, Kraft and organosolv, resulting in particle size, lignin source and dispersion processing being key parameters for adequate incorporation of lignin into the adhesive, which exhibited more than 50% better mechanical properties than non-lignin-based ones. In another study, it was demonstrated that both isocyanate and lignin source together with solvent were critical for adhesive strength performance [269]. Looking for synergistic effects, the combination of epoxy-modified lignin with PU emulsion also demonstrated significant enhancement of the adhesive properties of lignin-based adhesives [276].

### 4.5. Lignocellulose-Based Polyurethane Lubricating Greases

As already mentioned in previous sections, the future prospects of the lubricating grease industry rely on the discovery of suitable thickening agents and base oils that can replace the metal-based soaps and mineral oils usually utilised for those purposes, respectively (see Table 10).

In history, the use of natural materials as lubricating greases was widely carried out since the first mention of the topic, dating back to Ancient Egypt. Nonetheless, the discovery of metal-based soaps and mineral and synthetic oils crucially changed the course of lubricating grease production (see Table 11) [277].

In this sense, once more, natural resources can play a decisive role, providing suitable replacers for both main lubricating grease components, aiming to fulfil current industrial demands and enhancing biodegradation properties of lubricants based on non-renewable resources. Via polyurethane formation, structural networks able to withstand and thicken oils can be obtained, making them suitable for lubricating purposes. Some of the most used natural sources for PUs formation will be discussed in the following sections regarding their application as lubricating greases.

Both barley and wheat straws were considered for lubricating grease production by biological modification with *Streptomyces*, demonstrating excellent rheological and tribological properties. The straw origin once more was crucial, as the *Streptomyces* activity was extremely dependent on the biosource, producing completely different effects on the rheological behaviour. Friction coefficients of around 0.09 (around 0.02 lower than commercially available lithium-based lubricating grease formulations) were obtained [279]. Likewise, some of the main constituents, i.e., cellulose and lignin, have also been reported to be used as polyurethane-type thickening agents.

#### 4.5.1. Cellulose-Based Polyurethane Lubricating Greases

Several studies have taken advantage of the polyol structure of cellulose and its derivatives for the production of lubricating greases. Thus, PU formulations proposed as bio-based lubricating greases were obtained by functionalisation of methylcellulose with hexamethylene diisocyanate (HDI) and further mixing with castor oil. The different concentrations and methylcellulose/HDI ratios led to tunable characteristics, covering a wide range of lubricating grease consistencies [280,281]. Other studies have tested α-cellulose, 2-hydroxyethyl cellulose, methyl 2-hydroxyethyl cellulose and cellulose acetate propionate as precursors for PU-based lubricating grease formulations. The results let one conclude that the balance and size of the polar and non-polar groups turned out to be crucial in order to predict the rheological behaviour of the bio-based PUs [282]. Nonetheless, less purified cellulose-based systems as industrial cellulose pulps were also evaluated as an oil thickening agent, providing a more economical and industrial approach. Therefore, both *Eucalyptus globulus* and *Pinus radiata* cellulose pulps were assessed, the first one with two different purity grades, obtained by semi-mechanical cooking and commercial Kraft process. Cellulose pulps obtained from the soda pulping process of previously biologically treated wheat and barley straws with different *Streptomyces* strains were also targeted as thickening agents via PU formation, in this case by following a greener one-step approach. The modifications produced by the enzymatic activity of these strains were successfully correlated to the rheological properties of the lubricating greases [283]. All of them exhibited gel-like rheological characteristics with well-developed plateau regions within the frequency range studied, and microstructures very similar to those found in commercial lithium lubricating greases [284]. On the other hand, the tribological properties of many of these systems were also evaluated, as well as the mechanical stability, showing that most of them had suitable values for being considered potential replacers of traditional petroleum-based lubricating greases [283,284,285].

#### 4.5.2. Lignin-Based Polyurethane Lubricating Greases

Lignin, as shown in previous sections, can easily act as a lubricant additive in composites and other materials, in both liquid and solid lubricants. Nonetheless, in this section, those studies related to the production of lignin-based PUs with lubricating properties are summarised. Thus, Ma et al. [286] developed lignin-based PUs, with lignin acting as an emulsifier for [BMIm]PF6 ionic liquid lubricant. The tribological results demonstrated very low friction coefficient and wear scars, as well as excellent thermal stability. Several studies concerning lignin-based PU formulations acting as gelling agents in castor oil media with promising applications as lubricants have also been reported. Thus, in a preliminary study, several diisocyanates were tested, showing HDI having the best properties to act as a crosslinker, resulting in rheological characteristics similar to those observed for commercial lithium-soap-based lubricating greases [287]. In a greener approach, a one-step method for the preparation of lignin-thickened lubricating greases, free of solvents or catalysts, was carefully addressed (evaluating temperature, agitation speed, etc.), it being possible to obtain proper formulations not only with HDI but also with toluene diisocyanate (TDI), at room temperature and relatively low agitation speed [288]. The thickening potential of a modified lignin fraction derived from the enzymatic activity of *Streptomyces* strains in wheat straw was also evaluated. In this sense, the−OH group concentration increase due to the enzymatic digestion of wheat straw led to higher viscoelastic functions of the obtained oleogels [289]. Alternatively, the direct use of the laccase enzyme to modify lignin was also aimed at, producing significant differences between commercial- and residual lignin-based lubricating greases [290]. Furthermore, several residual lignin fractions from bioethanol or Kraft pulping processes of different species were valorised, exhibiting good properties as lubricant thickeners but dependent on the lignin origin, composition and extraction method [291,292,293]. Some of these formulations were tested for biodegradability and ecotoxicity, showing generally that there is no harm or danger to their disposal [294]. On the other hand, lignin-based lubricating greases formed with a polyurea-polyurethane thickener have also been patented lately [295]. Moreover, other patented lubricants also include lignin as a dispersing agent and polyurethane as a polymeric binder in their formulations [296].

### 4.6. Lignocellulose-Based Polyurethane Elastomers

The substitution or at least minimisation of the use of petroleum-based sources by the inclusion of bio-based plant-derived components in PU elastomers is considered in this section. Different types of natural vegetable sources have been reviewed and discussed hereunder.

Lignocellulose-based PU elastomers have been widely studied due to their low cost, high availability, biodegradability and excellent performance. Once more, the properties of the related elastomers have shown great dependency on origin, composition, growth conditions, etc. Nonetheless, the lack of industrial advance in the topic relies on incompatibilities related to differences in hydrophilicity, compromising suitable dispersion [297].

There are several lignocellulosic sources that have been analysed in literature for this purpose. For instance, sawdust has been tested for PU elastomeric formulations. Thus, acoustic panels with soundproof properties have been obtained from the combination of some components, sawdust and PU elastomer binder being two of them [298]. A close material, wood flour, was also used for composite formation with elastomeric properties, which was prepared in filaments by 3D-printing technology. The tensile strength exhibited a minimum when wood flour concentration varied between 0 and 40%, while elongation at break was seriously compromised by increasing concentration [299]. In another study, Mengeloğlu and Çavuş [300] tested different lignocellulose sources as fillers, such as teak wood flour and rice husk, which were previously milled and screened before being used. The results showed that a 15% filler concentration potentially increased mechanical properties, while a 30% concentration caused a dramatic drop in the already mentioned behaviour. In general terms, the performance of teak-based systems was slightly better than that of rice husk. By using rice straw, the liquefied products from it have also led to the formation of polyurethane elastomers [301]. Following this tendency, straw liquefied products were also used by Zhao et al. [302] to perform PU elastomers where special attention to the obtaining procedure and its relation to mechanical performance were paid. In the following sections, the use of cellulose, hemicellulose and lignin in PU elastomeric formulations is discussed in more detail.

#### 4.6.1. Cellulose-Based Polyurethane Elastomers

Cellulose and cellulose-derived materials have been known to act as proper fillers and endow materials with thorough properties, due to the fibrous structure and hydrogen bonding possibilities. Regarding PU elastomers, the same can be applied; nonetheless, the use of micro and nanocellulose has been more lately targeted. For instance, microfibrillated cellulose has been leveraged as casting material for PU formation, thus acting as tough elastomers for composite reinforcing [303]. The addition of up to 5 wt.% of microcrystalline cellulose to a PU based on MDI, butanediol and polytetramethylene glycol turned out a better performance of more than 650% tensile strength compared to the reference system [304]. Pei et al. [305] demonstrated that by incorporating cellulose nanocrystals in an MDI-poly(tetramethylene glycol)-based PU only at 1 wt.% concentration, stress at break could be 8-fold increased. In another work, the use of cellulose nanowhiskers prepared from microcrystalline cellulose allowed the formulation of shape-memory PU elastomers, triggered by water [306]. The addition of cellulose nanocrystals also demonstrated the improvement of the rheological properties of PU elastomers, with an increase of several orders of magnitude of the still unreacted suspensions, due to strong interactions between the nanocrystals and the polyol [307]. In another study, Saralegi et al. [308] developed from elastomeric to rigid bio-based PUs by increasing cellulose nanocrystal concentration. Interestingly, Lee et al. [309] incorporated cellulose nanofibres to a PU-urea matrix and reported a decrease in the elastic modulus by increasing cellulose concentration up to 1% and a further increase at 2%. Nonetheless, the mechanical properties were anyway enhanced. In castor oil/PEG-based PUs, the inclusion of cellulose nanocrystals obtained from *Eucalyptus globulus* was likewise positive for mechanical properties enhancement, reporting shifts from 5 to 12 MPa and from 1 to 5 MPa of tensile strength and Young modulus respectively, in comparison with the cellulose-free system [310]. 

#### 4.6.2. Hemicellulose-Based Polyurethane Elastomers

Only a few studies proposed either hemicellulose derivatives or the monomers that comprise its structure as suitable polyols for the preparation of PU elastomers. Glucomannan was used in different concentrations to tune castor-oil-based PU properties from elastomeric to very rigid systems within the range of 5–90 wt.% concentration [311,312]. In a related work, films with elastomeric properties were elaborated by modifying glucomannan molecular weight [313]. It is also well known that very interesting furan derivatives can be obtained from hemicellulose, like furfural. Such compounds have also been used as sources for polyurethane formation, some of them serving as elastomers [83].

#### 4.6.3. Lignin-Based Polyurethane Elastomers

Lignin and lignin derivatives have also been occasionally used as polyols and fillers for elastomers production [314]. One of the most important derived chemicals that can be obtained from lignin is vanillin. Apart from the already mentioned uses in previous sections, PU elastomers have likewise been generated. Vanillin inclusion propelled mechanical properties, causing Young modulus and strain at break to increase up to around 130 and 150% respectively [315]. Another lignin derivative that has been used as a copolymer in PU elastomers is lignin-derived polycarboxylic acid. With content of only 2.5%, the Young modulus and tensile stress at different strains (100 and 300%) were augmented 384, 135 and 90% respectively [316].

In the development of lignin-based PU elastomers, frequently lignin acted as polyol, becoming a structural part of the formulation, while sometimes it is only cast or mixed, acting as filler. As examples of the first case, improved thermal and mechanical properties were reported by the use of unmodified lignin as polyol along with poly (propylene glycol) and TDI as a crosslinker, yielding PUs with outstanding tensile performance (176 MPa, 33 MPa and 1394% of Young modulus, tensile strength and elongation at break respectively) by the addition of up to 40% lignin. In addition, the better performance of low-molecular-weight lignin fractions was reported [317]. The use of acetylated lignin and polyethylene glycol (PEG) as copolymers allowed elongations at break higher than 2000% to be obtained [318]. Likewise, PEG with oxidised lignin was used by Zhang et al. [319], resulting once more in great tensile performance. On the other hand, Liu et al. [320] took advantage of enzymatic procedures to alter lignin structure, which decreased molecular weight, thus imparting stronger mechanical properties to the network, i.e., up to 60 MPa tensile strength. Elastomers with excellent cushioning properties which could well act as coatings or shoe soles were likewise obtained by the inclusion of lignin in castor-oil-based PUs, showing an outstanding 88-fold enhancement at the stress at break in compressive performance, along with an improvement of the strain at failure from 50 to 93%, compared to the lignin-free system. The tensile properties were also enhanced by 17- and 7-fold regarding the Young modulus and stress at break, respectively. The dynamic properties of these elastomers were also tested, showing excellent results even for high loads and long-time essays [245]. Using lignin as filler, Ciobanu et al. [321] performed PU elastomers based on poly(ethyleneadipate), ethylene glycol, and MDI where lignin was cast using DMF as solvent at concentrations from 4 to 23%. The results indicated, once more, improved performance on mechanical properties with only a 10 wt.% lignin concentration, where the tensile strength and elongation at break were augmented up to 370 and 160% respectively. Acting as filler, lignin is usually responsible for enhancing mechanical properties, such as Young modulus or compressive performance [173,241,322]. In general, the higher the lignin concentration, the higher both the storage and Young moduli up to a certain concentration depending on the formulation, since lignin acts as a rigid component [241]. In this sense, storage modulus has been raised 6-fold by the addition of up to 60% lignin to polypropylene-glycol-based PUs [323]. In another study, Ortíz-Serna et al. [247] evaluated both the thermal and mechanical properties of Kraft and organosolv lignin acting as fillers for PU elastomers based on castor oil and butanediol, exhibiting great differences between these products. Culebras et al. [324] tested Alcell organosolv and hydroxypropyl-modified Kraft hardwood lignin for the same purpose, being the first one able to achieve excellent mechanical properties, with Young modulus of 80 GPa, comparable to commercial elastomers. Other authors tested other different lignins, such as wheat straw soda-derived, Indulin AT softwood kraft, Protobind 1000 soda, corn stover or sulphite hardwood lignins [241,325]. Frigerio et al. [326] found particle size, polar surface and agglomeration tendency to be key points in lignin performance as filler. Non-modified lignin was demonstrated to carry out the functions of both crosslinker and reinforcing agent in silicone-based PUs, matching some of the properties of commercially available silicone PUs [327]. Some of these elastomers can act as sealants, materials in surfboards, car interiors, household items and sporting indoor courts [328].

**Figure 4:**
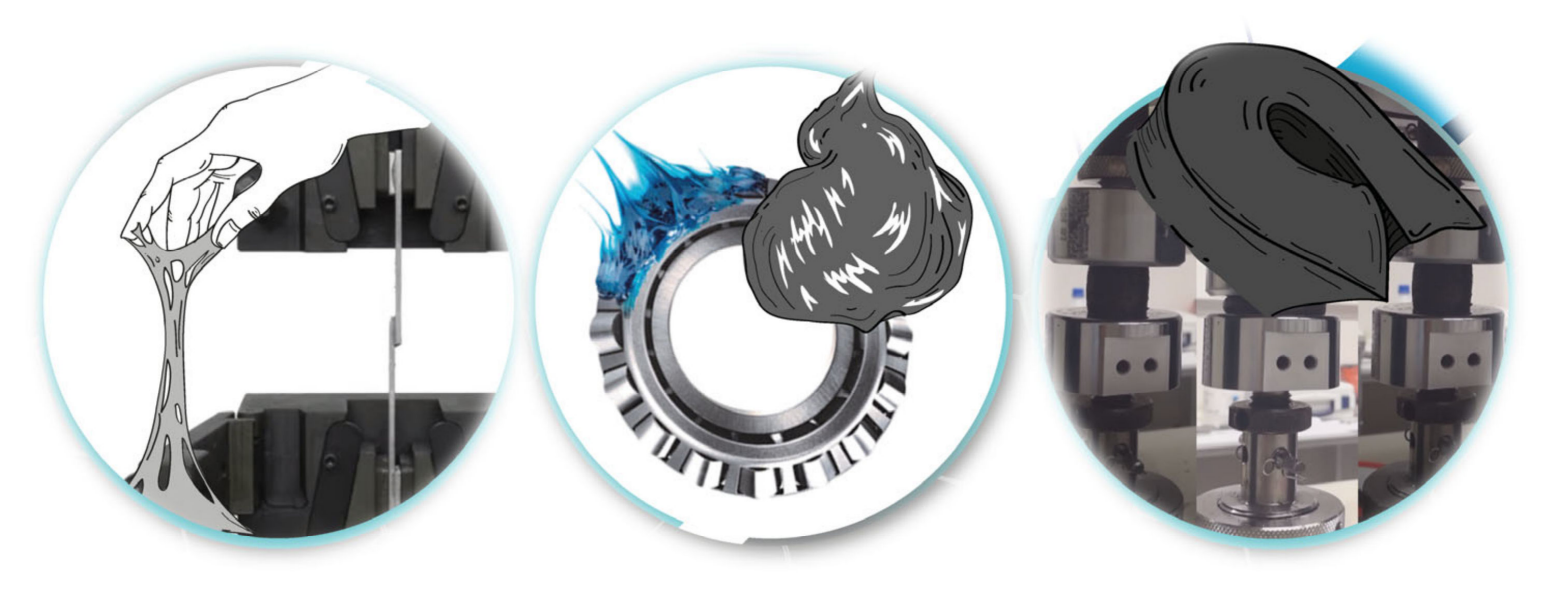
Main bio-polyurethanes studied, i.e., adhesives, lubricating greases and elastomers, from left to right, with testing or application as background.

Finally, the three main bio-polyurethane types studied, i.e., adhesives, lubricating greases and elastomers, have been included in Figure 4, with the background showing biomaterial testing or application.

## 5. Conclusions

Due to emerging environmental concern during the last few decades, lignocellulose is playing a fundamental role in research. Either by its use as a whole, fractionated into its main biopolymers or by the derived production of small molecules, great focus is being paid due to its environmentally friendly alternative and ubiquitous production. On the other hand, the residual character of some lignocellulose-derived fractions boosts possibilities for their valorisation, especially lignin. The present review highlights the crucial importance of the study of the lignocellulose extraction, separation and decomposition, and provides an insight into these modifications, mainly based on thermal, mechanical, chemical and biological pretreatments. Special attention is paid to biological processes whether by the action of fungi or bacteria, due to the greener and more environmentally friendly characteristics. By selecting a determined type of fungi or bacteria, some of the fractions may remain unaltered, while others may be decomposed. Therefore, the possibility to obtain different final products becomes massive, depending also on the biomass selected. 

Furthermore, this review focusses its efforts on the use of lignocellulose, fractionated or not, in the production of biofuels, biochemicals and biomaterials. Thus, a wide range of biofuels, from the well-known biodiesel, biogas and bioethanol to other emerging species like dimethyl ether, biohydrogen, biofene®, etc., have been produced. Moreover, the last technologies regarding genome-editing approaches and biological procedures are presented. Considering biochemicals, an extremely wide range of products has been analysed and obtained in literature, and here we collect and separate them into those obtained from cellulose, hemicellulose and lignin. Biomaterials from lignocellulose have also been targeted massively lately, and many different products, like films, coatings, membranes, hydro-, oleo- and aero-gels, emulsifiers, fillers, flocculants, etc. have been produced, with many different applications, in fields such as transportation, textile, packaging, environmental remediation, plastic replacers, house and leisure items, health and hygiene, etc. Among the wide range of biomaterials, the emerging and outstanding characteristics of polyurethanes have attracted the attention of this review, especially in fields that are not usually deeply considered, such as adhesion, grease lubrication and elastomeric cushioning. Therefore, we have reported high-performance alternatives to traditional petroleum-derived products in those areas, such as adhesives that substantially exceed the adhesion performance of commercially available formulations in different surfaces, lubricating greases with tribological behaviour superior to that of lithium and calcium soap-based lubricants, and elastomers with excellent static and dynamic performance.

Overall, this review provides an extensive overview of the current state-of-the-art of the pretreatments for lignocellulosic materials, and the development of lignocellulose-derived biofuels, biochemical and biomaterials, with special attention to PU-based formulations for adhesion, lubricating greases and elastomeric cushioning fields.

## Figures and Tables

**Table 1:** Composition of representative lignocellulosic feedstocks [12].

Feedstocks	Carbohydrate Composition (% Dry Weight)		
	Cellulose	Hemicellulose	Lignin
Barley hull	24	36	19
Barley straw	36–43	24–33	6.3–9.8
Bamboo	49–50	18–20	23
Banana waste	13	15	14
Corn cob	32.3–45.6	39.8	6.7–13.9
Corn stover	35.1–39.5	20.7–24.6	11.0–19.1
Cotton	85–95	5–15	0
Cotton stalk	31	11	30
Coffee pulp	33.7–36.9	44.2–47.5	15.6–19.1
Douglas fir	35–48	20–22	15–21
Eucalyptus	45–51	11–18	29
Hardwood stems	40–55	24–40	18–25
Rice straw	29.2–34.7	23–25.9	17–19
Rice husk	28.7–35.6	11.96–29.3	15.4–20
Wheat straw	35–39	22–30	12–16
Wheat bran	10.5–14.8	35.5–39.2	8.3–12.5
Grasses	25–40	25–50	10–30
Newspaper	40–55	24–39	18–30
Sugarcane bagasse	25–45	28–32	15–25
Sugarcane tops	35	32	14
Pine	42–49	13–25	23–29
Poplar wood	45–51	25–28	10–21
Olive tree biomass	25.2	15.8	19.1
Jute fibres	45–53	18–21	21–26
Switchgrass	35–40	25–30	15–20
Winter rye	29–30	22–26	16.1
Oilseed rape	27.3	20.5	14.2
Softwood stem	45–50	24–40	18–25
Oat straw	31–35	20–26	10–15
Nut shells	25–30	22–28	30–40
Sorghum straw	32–35	24–27	15–21
Tamarind kernel powder	10–15	55–65	-
Water hyacinth	18.2–22.1	48.7–50.1	3.5–5.4
Miscanthus *	37	29	10
Oat husks **	23	35	25

* Adapted from [15]. ** Adapted from [16].

**Table 2:** Approximate percentages of linkages found in softwood and hardwood lignin [33].

Linkage Type	Approximate Percentage (%)	
	Softwood	Hardwood
β-O-4	45–50	60
5–5	18–25	5
β-5	9–12	6
4-O-5	4–8	7
β-1	7–10	7
β-β	3	3

**Table 3:** Enzymes involved in the hydrolysis of complex heteroarabinoxylans.

Enzyme	Objective
Endo-β-l,4-xylanase	β-1,4-xylose linkage hydrolysation of the xylan backbone
Exo-xylanase	β-1,4-xylose linkage hydrolysation, releasing xylobiose
β-Xylosidase	Release short-chain xylooligosaccharides and xylose from xylobiose
α-L-Arabinofuranosidase	Terminal non-reducing α-arabinofuranose hydrolysation from arabinoxylans
α-Glucuronidase	Release glucuronic acid from glucuronoxylans
Acetylxylan esterase	Acetylester bond hydrolysation in acetyl xylans
Ferulic acid esterase	Feruloylester bond hydrolysation in xylans
ρ-Coumaric acid esterase	ρ -coumaryl ester bond hydrolysation in xylans

**Table 4:** Aromatic products detected from lignin breakdown [51,65,66].

Compound		Fungal Lignin Degrader	Bacterial Lignin Degrader
Benzoic acid	4-hydroxy	*P. chrysosporium*	*A. aneurinilyticus*, A. sp. 75iv3
	4-hydroxy-3-methoxy	*P. chrysosporium*	*A. aneurinilyticus*
	4-hydroxy-3-methoxy-6-carboxy	*P. chrysosporium*	
	4-hydroxy-3-methoxy-5-carboxy		*P. putida, R. jostii* RHA1
	3,4-dimethoxy	*P. chrysosporium*	
	3,4-dimethoxy-2-carboxy	*P. chrysosporium*	
	2-hydroxy-3-methoxy		*A. aneurinilyticus, P. putida*
	2,3-dihydroxy		
	2,3,4-trihydroxy		*Bacillus sp.*
Benzaldehyde	4-hydroxy-3-methoxy		*S. paucimobilis*
	3,4,5-trimethoxy		*Bacillus sp.*
Cinnamic acid	4-hydroxy		*Bacillus sp.*
	4-hydroxy-3-methoxy		*Bacillus sp., P. putida, R. jostii* RHA1
Biphenyl-5,5′-dicarboxylic acid, 2,2′-dihydroxy, 3,3′-dimethoxy	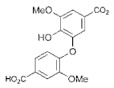	*P. chrysosporium*	
Diphenyl ether		*P. chrysosporium*	
Propiophenone-3′-hydroxy	4-hydroxy-3-methoxy		*S. paucimobilis. P. putida, R. jostii* RHA1
Acetophenone	4-hydroxy-3-methoxy		Soil metabolite
Phenol	2-methoxy		Soil metabolite
	2-methoxy-4-vinyl		Soil metabolite
Vanillin			*R. jostii* RHA1, *A*. sp. 75iv3

**Table 5:** Comparison of the different pretreatment methods used for lignocellulosic degradation methods. Good or bad performance regarding cost, toxic byproduct formation and applicability are marked by 🗸 and ×, respectively [61].

Pretreatment	Cost	Toxic Byproduct	Applicable to a Wide Range of Biomass	Remarks
Acid pretreatment	🗸	×	🗸	Inhibitors limited by dilute acid use
Freezing	🗸	🗸	×	Freezing/thawing cycles
Milling	🗸	🗸	🗸	Used for bioethanol and biogas production
Liquid hot water	🗸	🗸	×	High water and energy inputs
Organic solvent (Organosolv)	×	×	🗸	Low boiling point of the solvent. Solvent recycling is required
Oxidation	×	🗸	🗸	High cost of ozone generation. Ozone handling is required
Steam explosion	×	×	🗸	High cost of steam generation
Extrusion	🗸	🗸	🗸	Hydrolysis efficiency is improved
Wet oxidation	×	🗸	×	Less water use as no washing is required
CO_2_ explosion	×	🗸	🗸	High cost for pressure maintenance
Microwave irradiation	×	🗸	🗸	More effective than conventional heating
Ultrasound	×	🗸	🗸	Low temperature and time required
Ammonium fibre expansion	×	🗸	🗸	Less effective for biomass with high lignin contents
Ionic liquid	×	🗸	🗸	Stability and reuse. Instability may cause contamination
Biological pretreatment	🗸	🗸	🗸	Increases delignification. Able to reduce polymerisation
Hydrothermal liquefaction	×	🗸	🗸	Lignocellulosic materials are depolymerised into bio-oil, biogas, biochar and water-soluble compounds

**Table 6:** Main biofuels, the associated energy and corresponding processing routes reported in the literature.

Biofuel	Lower Heating Value	References
Biodiesel	32.6 MJ/L	[76]
Bioethanol	21.2 MJ/L	[76,77]
Biocrude	35.0 MJ/kg	[76]
Bio-oil	40 MJ/kg	[76,78]
Biogas	13–17 MJ/m^3^	[71,79]
Biohydrogen	13 MJ/m^3^	[71,79]
Biobutanol	27.8 MJ/L	[80]

**Table 7:** Main building blocks and their derivatives obtained from cellulose and hemicellulose [84].

Compound	Production	Derived Products
Succinic, fumaric and malic acid	Biofermentation	Tetrahydrofuran (THF), 1,4-butanediol, 2-pyrrolidone, o-butyrolactone, N-methyl-2-pyrrolidone (NMP)
2,5-Furan dicarboxylic acid	Chemical (oxidative dehydration of glucose) and biological	(2,5-Bis(aminomethyl)-tetrahydrofuran, 2,5-dihydroxymethyl-tetrahydrofuran, 2,5-dihydroxymethyl-furan
3-Hydroxy propionic acid	Biofermentation	1,3-Propanediol, acrylic acid, acrylamide
Aspartic acid	Chemical and biological pathways	2-Amino-1,4-butanediol, aspartic anhydride, 3-aminotetrahydrofuran, amino-γ-butyrolactone
Glucaric acid	Chemical (starch oxidation by nitric acid or bleach)	Glucaro-γ-lactone, polyhydroxypolyamides, glucarodilactone, glucaro-δ-lactone
Glutamic acid	Biofermentation	Glutaminol, glutaric acid, norvoline, 1,5-pentandiol, 5-amino-1-butanol
Itaconic acid	Chemical and biofermentation	3-Methylpyrrolidine, 3- & 4-methyl NMP, 3-methyl THF, 2-methyl-1,4-butanediol.
Levulinic acid	Chemical (acid decomposition of six-carbon sugars)	Diphenolic acid, 2-methyl-THF, b-acetylacrylic acid, 1,4-pentanediol
3-Hydroxybutyrolactone	Chemical (oxidative degradation of starch)	3-Hydroxytetrahydrofuran, 3-aminotetrahydrofuran, acrylate-lactone
Glycerol	Transesterification (via chemical or biological pathways)	Glyceric acid, 1,3-propanediol, propylene glycol
Sorbitol	Chemical (glucose hydrogenation)	Isosorbide, propylene glycol, ethylene glycol, 1,4-sorbitan
Xylitol/arabinitol	Chemical (hydrogenation of xylose and arabinose) and biological	Xylaric acid, propylene glycol, ethylene glycol, lactic acid

**Table 8:** Main procedures from lignin depolymerisation along with main products obtained.

Depolymerisation	Procedures	Products	Refs
Non-reductive depolymerisation	Thermal, hydrothermal, oxidative, acid and base catalysed, solvolytic	Vanillin, syringaldehyde, acetosyringone, guaiacylacetone, p-hydroxylated phenol acetovanillone, syringol, guaiacol, phenol, catechol, alkylcatechols, creosol, p-hydroxybenzaldehyde, vanillic, protocatechuic, syringic, homovanillic and p-hydroxybenzoic acid, aliphatic carboxylic acids (succinic, acetic and formic acid)	[81,86]
Reductive depolymerisation	Hydroprocessing, liquid phase reforming	Cresol, xylenol, phenol with long alkyl chains, p-substituted methoxyphenols,	[86]

**Table 9:** Glucomannan-based materials, characterisation performed and future application prospects in various fields [236].

Components	Potential Applications
Chitosan/Konjac glucomannan (KGM)	Membrane with superior dehydration
KGM/Chitosan	Food industry, biomaterial matrix, biomedical material
KGM/Ethyl cellulose	Films for food packaging
Glucomannan–Chitosan–Nisin	Active packaging material
KGM/Gellan gum	Food packaging material
KGM/Poly(acrylic acid)	Specific drug delivery
KGM/Polyacrylamide/Sodium xanthate	Hydrogels for drug delivery
KGM/poly(methacrylic acid)	Specific drug delivery
KGM/Polyvinyl alcohol	Pervaporation dehydration, food package film
KGM/Xanthan gum	Gels for delivery systems, specific drug delivery
KGM/Alginate/Chitosan	Controlled release
KGM/Carboxymethyl cellulose	Emulsion stabiliser
KGM/Curdian	Food films and coatings
KGM/Poly(aspartic acid)	Carrier for drug delivery
KGM/Cellulose	Separation
KGM/Whey protein	Edible food films
KGM/Sodium alginate	Food films
KGM/Gelatin	Specific drug delivery
KGM/Starch	Edible food films & coatings
KGM/Poly(diallydimethylammonium chloride)	Antibacterial in biomedicine
KGM/xanthan gum	Drug delivery
KGM-graft-Polyacrylamide-co-sodium xanthate	Flocculant
KGM	Coating

**Table 10:** Most-used base oils and thickeners for lubricating greases.

Base Oils	Thickeners
Mineral oil	Sodium soap
Synthetic oil	Calcium soap
Diester	Lithium soap
Silicone liquid	Aluminium soap
Phosphate ester	Lithium complex
Fluorinated silicone	Calcium complex
Chlorinated silicone	Aluminium complex
Polyglycol	Bentonite
Castor oil	Silicon oxide
	Carbon/graphite
	Polyurea
	Polyethylene
	Indanthrene dye
	Phthalocyanine dye

**Table 11:** Lubricating grease evolution through history [277,278].

Date	Event
About 1400 BC	The use of animal fat and limestone for lubrication of axes of Hittite chariots was started. In the same period, lubricants based on olive oil and limestone were used in Ancient Egypt
1845	A lubricant consisting of mineral oil, animal fat and limestone was invented in the United States
1853	The first sodium lubricant based on beef fat appeared in the United Kingdom
1912	The production of calcium lubricants with the dispersion medium based on mineral oil was started in Japan
1938	Lithium lubricants were developed. They quickly conquered the world and were used as multipurpose lubricants
1954	Invention of complex aluminium lubricants for operation at high temperatures
1955	Invention of urea greases in the United States
1960–Present	Resurgence of vegetable-oil-based lubricating greases

## Data Availability

Not applicable.
